# Postprandial lipemic response in dairy-avoiding females following an equal volume of sheep milk relative to cow milk: A randomized controlled trial

**DOI:** 10.3389/fnut.2022.1029813

**Published:** 2023-01-04

**Authors:** Fei Teng, Linda M. Samuelsson, Amber Marie Milan, Arvind Subbaraj, Michael Agnew, Aahana Shrestha, David Cameron-Smith, Li Day

**Affiliations:** ^1^AgResearch Ltd., Grasslands Research Centre, Palmerston North, New Zealand; ^2^The Liggins Institute, The University of Auckland, Auckland, New Zealand; ^3^AgResearch Ltd., Lincoln Research Center, Lincoln, New Zealand; ^4^Riddet Institute, Palmerston North, New Zealand; ^5^College of Health, Medicine and Wellbeing, University of Newcastle, Callaghan, NSW, Australia

**Keywords:** ovine milk, bovine milk, lipid digestion, milk alternative, fatty acids, postprandial lipemia, adult nutrition, lipidomics

## Abstract

**Background:**

Sheep milk (SM) is an alternate dairy source, which despite many similarities, has both compositional and structural differences in lipids compared to cow milk (CM). Studies are yet to examine the apparent digestibility of SM lipids, relative to CM, and the potential impact on the plasma lipidome.

**Objective:**

To determine the response of the circulatory lipidome to equal volume servings of SM and CM, in females who avoid dairy products.

**Method:**

In a double-blinded, randomized, cross-over trial, self-described dairy avoiding females (*n* = 30; 24.4 ± 1.1 years) drank SM or CM (650 mL; 33.4 vs. 21.3 g total lipid content; reconstituted from spray dried milk powders) following an overnight fast. Blood samples were collected at fasting and at regular intervals over 4 h after milk consumption. The plasma lipidome was analyzed by LC-MS and fatty acids were quantified by GC-FID.

**Results:**

The overall postprandial triglyceride (TG) response was similar between SM and CM. TG concentrations were comparable at fasting for both groups, however they were higher after CM consumption at 30 min (interaction milk × time *p* = 0.003), well before any postprandial lipemic response. This was despite greater quantities provided by SM. However, there were notable differences in the postprandial fatty acid response, with SM leading to an increase in short- and medium-chain fatty acids (MCFAs) (C6:0, C8:0, and C10:0) and several long-chain fatty acids (LCFAs) (C18:1 *t*11, *c*9, *t*11-CLA, and C20:0; interaction time × milk *p* < 0.05). This corresponded to a greater postprandial response for medium chain triglycerides (MCTs) C10:0, including TG(10:0/14:0/18:1), TG(16:0/10:0/12:0), and TG(16:0/10:0/14:0) (interaction time × milk *p* < 0.05).

**Conclusions:**

Despite a higher fat content, SM ingestion resulted in a greater circulating abundance of MCTs, without increasing total postprandial triglyceride response, when compared to CM. The greater abundance and postprandial appearance of MCTs may provide advantageous metabolic responses in children and adults.

**Unique identifier and registry:**

U1111-1209-7768; https://www.anzctr.org.au/Trial/Registration/TrialReview.aspx?id=375324.

## Introduction

Avoidance of dairy products is evident globally (1, 2), where perceived or actual intolerance to bovine (cow) milk (CM) (3) impacts consumption behaviors (4, 5). Dairy products are recognized as an important source of nutrients (6), including calcium and high-quality protein, which are essential for growth and development. Milk and dairy products are also an important source of dietary lipids, although there is continued debate on the impact of these lipids on cardiometabolic health risks (7). One component of this issue's complexity is that dairy is not only rich in potentially pro-atherogenic saturated fatty acids (SFAs) (8–10), but it also contains a wide range of other lipid species, such as saturated medium-chain triglycerides (MCTs), which comprises up to ~15% of total lipids (11). In contrast to long-chain triglycerides (LCTs), MCTs undergo preferential hydrolysis to free fatty acids (FFAs) and are absorbed directly into the portal vein, and hence transported rapidly to the liver for oxidation (12). Therefore, these lipid species have minimal impact on circulatory lipoproteins and other atherogenic risk factors (13, 14).

Sheep (ovine) milk (SM), is an alternate ruminant milk which maintains many of the key nutritional features of CM. However, there are differences between the two milk types: for example SM proteins are more digestible than CM proteins, and provides a greater quantity of branched-chain amino acids (BCAAs) with a corresponding elevation of the postprandial circulating BCAA response (15). Despite a higher energy and nutrient content in SM, SM did not increase adverse digestive symptoms relative to CM, and reduced the breath H_2_ response in dairy-avoiding females, as reported previously (16). Compared to CM, SM has higher proportions (14.5–27.6 vs. 10.7–16.8%) of short- and medium-chain fatty acids (SCFA; MCFA) (17–19). Additionally, higher proportions of these MCFAs are in the *sn*-1/3 positions (48.3–67.3 vs. 12.8–47.1%) of triglycerides (TGs) in SM compared to CM (20, 21). These compositional and structural characteristics contribute to the better digestibility of SM compared with CM, higher release of MCFAs and polyunsaturated fatty acids (PUFAs) and lower release of saturated fatty acids (SFAs) according to our previous study using an *in vitro* model (20, 22). These results highlighted that fat content is not necessarily the sole determinant of nutritional quality, but rather that the compositional and structural differences in lipids can provide beneficial properties. Therefore, it is important to investigate the impact of SM on postprandial lipemia in human clinical studies due to the limitations of the previous *in vitro* study such as the specificity of human digestive enzymes and the impact of protein in fat metabolism.

The aim of this study was therefore to compare, on a “portion-for portion” basis, the appearance of circulating fatty acids following ingestion of SM or CM, and further to compare the appearance of other lipid species [i.e., TGs, phospholipids (PLs), sphingomyelin (SphM), etc.] in circulation. Spray dried powdered milk was selected as a shelf stable milk suitable for both SM and CM. To improve the generalizability of the results, reconstituted SM powder from two different producers and commercially available reconstituted CM powder were used as the test drinks in this study.

We hypothesized that consistent with the compositional differences, concentrations of circulating SCFAs and MCFAs would be greater following the ingestion of SM compared to CM.

## Methods

### Experimental design

The double-blinded, cross over randomized controlled trial was conducted at the Liggins Institute, The University of Auckland between July and November 2018. The primary outcome of the study has been reported by Milan et al. (15). Secondary outcomes of lactose malabsorption and digestive comfort have also been reported by Shrestha et al. (16). The additional secondary outcome of lipid responses was assessed and is reported here. The study was conducted according to the guidelines laid down in the Declaration of Helsinki and all procedures involving human subjects were approved by the New Zealand Health and Disability Ethics Committees (Reference No. 18/NTB/92). The trial was prospectively registered with the Australian New Zealand Clinical Trials Registry (ACTRN12618001030268). Written informed consent was obtained from eligible participants prior to the study commencement.

A total of 32 healthy young women aged 20–40 years with BMI 18–28 kg/m^2^ were recruited using digital and printed advertisements. Two subjects withdrew prior to the completion of the study and were excluded from further analyses. All participants self-reported dairy avoidance. Subjects with known dairy allergy, current or history of gastrointestinal, cardiovascular, or metabolic disease, consuming medications expected to interfere with normal digestive and metabolic processes like proton pump inhibitors, antibiotics, or prebiotics (3 months prior to the study) were not eligible. Participants with pre-existing cardiovascular diseases or self-reported alcohol intake >28 units per week were also excluded.

### Study procedures

Eligible participants were randomized to consume 650 mL of either SM or CM test drinks, which were made from reconstituted whole milk powders, on two occasions at least 1 week apart. Milk test drinks were prepared from milk powder the evening prior to the visit using heated (30°C) filtered water, shaken vigorously, then stored over night at 4°C. Pre-weighed portions of sheep or cow milk powder (98 or 81 g, respectively) were reconstituted in 585 mL water to provide a total solid content equivalent to the respective lipid SM or CM. The reconstituted milk was served chilled. Randomization sequences were computer generated using www.randomizer.org. Both participants and investigators were blinded to the treatment identity and allocation was implemented through sealed envelopes. One day prior to the visits, subjects were advised to avoid vigorous physical exercise, dairy and fiber rich food, and were provided with a standardized low fat, low dietary fiber dinner after which they were to remain fasted from 10.00 p.m. Upon arrival the following morning, fasting blood samples were collected. Subjects then consumed 650 mL of milk within 10 min. Following milk ingestion, blood samples were collected every 30 min until 2 h and hourly thereafter until 4 h.

Venous blood samples were collected in EDTA containing vacutainers (Becton Dickinson and Company, Mount Wellington, New Zealand), and plasma was removed after centrifugation at 2,000×g for 15 min at 4 °C and frozen at -80°C prior to analyses.

Plasma cholesterol, triglycerides, high-density lipoprotein-cholesterol and low-density lipoprotein-cholesterol levels at baseline were measured using a Cobas c311 clinical chemistry analyzer (Roche Diagnostics, Manheim, Germany).

### Milk treatments

Whole SM powder was provided by Blue River Dairy Ltd. (batch no. F2125/HC08) and Spring Sheep Milk Co. (batch no. MAN: NOV17-JAN18) and these were blended 1:1 prior to weighing and reconstitution for both consumption and all subsequent milk analysis. Whole CM powder was commercially sourced from NZMP (New Zealand Milk Products, Fonterra Co-Operative Group, Auckland, New Zealand). Milk powders were stored at -20°C prior to use.

The volume of 650 mL was selected to align with previous studies investigating milk digestion dynamics (23), to provide a quantity of fat great enough to elicit a postprandial lipemic response [>20 g fat per serve (24)], and to provide a quantity of milk sufficient for the primary outcome of amino acid concentrations in circulation [>0.24 g/kg body weight of protein (25)], and the secondary outcome of lactose malabsorption [>250 mL milk (16)].

The proximate compositions per 100 mL of the sheep and cow milk test drinks are listed in Table 1. The reconstituted SM test drink had higher concentrations of proteins (29.9 vs. 19.4 g per 650 mL), total solids (91.7 vs. 79.0 g per 650 mL), total energy (2,140.4 vs. 1,649.3 kJ per 650 mL), and fat (33.4 vs. 21.3 g per 650 mL) but lower lactose (24.9 vs. 33.3 g per 650 mL) than CM (16). With a higher total protein content than CM, the total amino acid content of SM was also greater (50.6 vs. 31.6 mg/mL). Proportions of amino acids as % content were generally similar (within 5%), except for tryptophan (1.53 vs. 1.29%) and alanine (3.68 vs. 3.34%) which were present in 18% and 10% higher proportion in SM compared to CM, respectively (15).

**Table 1:** Proximate composition per 100 mL of the sheep and cow milk test drinks.

**Component**	**Cow milk**		**Sheep milk**	
	**Amount**	**%**	**Amount**	**%**
Total energy (kJ)	253.7	–	329.3	–
Fat (g)	3.3	(3.3)	5.1	(5.1)
Protein (g)	3.0	(3)	4.6	(4.6)
Lactose (g)	5.1	(5.1)	3.8	(3.8)
Total solids (g)	12.2	(12.2)	14.1	(14.1)
Solids non-fat (g)	8.9	(8.9)	9.3	(9.3)

Test drinks were prepared from 25 g whole cow milk powder or 30 g whole sheep milk powder reconstituted in 180 mL water, respectively, per a total reconstituted volume of 200 mL.

A total of 415 lipid species were identified in the cow and sheep milk test drinks. The number of species detected in each lipid class are detailed in Supplementary Table 1 and includes both neutral lipids [TGs and diglycerides (DGs)], and polar lipids [ceramides (Cer), lysophosphatidylcholines (LPC), phosphatidylcholines (PC), phosphatidylethanolaminse (PE), and sphingomyelins (SphM)]. Among these lipid species, 87.2% lipids were TGs (362 species). SM contained nearly 1.6-fold more fat than the CM test drink and most of the lipids were more abundant in SM (Supplementary Table 1). SM contained higher proportions of MCTs, monounsaturated TGs, polyunsaturated TGs and SphM, and lower proportions of saturated TGs, long-chain TGs, PC and PE. Previous work showed that TGs containing the short- and medium chain fatty acids (C4:0–C12:0) and the long-chain fatty acid C18:1 in the *sn*-1 or *sn*-2 positions are more highly abundant in SM, and the same is true for DGs containing C4:0, C8:0, C:10, and C12:0 (20). With regards to polar lipids, PC(32:0), PC(34:0), PC(34:1), PC(36:1), PE(18:1/18:2), and SphM(d32:0), SphM(d34:1), and SphM(d36:4) were more abundant in the sheep milk test drink.

In general, sheep milk contained higher concentrations of all fatty acids than cow milk, which is not surprising given the higher fat content of sheep milk (Table 2). In addition, the proportion of fatty acids differed between the two milk types: SM contained higher proportions of short- and medium- chain fatty acids (SCFAs; MCFAs, i.e., C4:0–C12:0), branched-chain fatty acids (BCFAs) and polyunsaturated fatty acids (PUFAs), while the proportion of long-chain fatty acids (LCFAs, ≥C12:0) was lower. Individually, both the abundance and proportions of C8:0 (0.98 mg/mL and 2.59%) and C10:0 (3.20 mg/mL and 8.46%) in SM were more than twice of that in CM (0.39 mg/mL and 1.21% for C8:0; 0.90 mg/mL and 2.80% for C10:0, Table 2). The proportions of BCFAs (i.e., *iso* C14:0, *anteiso* C15:0, *iso* C15:0, *iso* C16:0, *iso* C17:0, and *anteiso* C17:0) and unsaturated FAs such as C18:1 *t*9, C18:1 *t*11, and C18:2n6 were also higher in SM than CM. In contrast, C14:1 *c*9 (myristoleic acid), C16:0 (palmitic acid), C16:1 *c*9 (palmitoleic acid), and C18:3n3 (α-linolenic acid) were found in higher proportions in CM.

**Table 2:** Fatty acid composition of the sheep and cow milk test drinks^*^.

**FAs**	**Sheep milk**		**Cow milk**	
	**mg/mL**	**% total**	**mg/mL**	**% total**
		**FAs**		**FAs**
C4:0	1.35	3.56	1.12	3.50
C6:0	1.01	2.67	0.68	2.11
C8:0	0.98	2.59	0.39	1.21
C10:0	3.20	8.46	0.90	2.80
C10:1	0.09	0.25	0.09	0.27
C12:0	1.79	4.73	1.47	4.59
*iso* C14:0	0.05	0.12	0.03	0.11
C14:0	4.40	11.6	3.91	12.3
C14:1 *c*9	0.07	0.17	0.27	0.84
*anteiso* C15:0	0.20	0.52	0.15	0.47
*iso* C15:0	0.11	0.28	0.09	0.28
C15:0	0.44	1.15	0.33	1.05
*iso* C16:0	0.10	0.26	0.06	0.19
C16:0	9.77	25.7	10.2	32.1
C16:1 *c*9	0.32	0.83	0.36	1.14
*iso* C17:0	0.27	0.71	0.18	0.56
*anteiso* C17:0	0.15	0.39	0.12	0.36
C17:0	0.19	0.51	0.14	0.45
C17:1 *c*9	0.11	0.29	0.06	0.19
C18:0	3.15	8.30	3.14	9.83
C18:1 *t*9	0.08	0.22	0.04	0.12
C18:1 *t*10	0.12	0.33	0.00	0.00
C18:1 *t*11	0.91	2.38	0.72	2.24
C18:1 *c*9	6.10	16.1	5.11	16.0
C18:1 *c*11	0.24	0.63	0.16	0.51
C18:2n6	0.30	0.81	0.17	0.52
C18:3n3	0.15	0.39	0.16	0.51
C20:0	0.06	0.15	0.04	0.11
c9, *t*11-CLA	0.32	0.84	0.27	0.83
BCFAs	0.88	2.28	0.63	1.97
SCFAs	2.36	6.23	1.80	5.61
MCFAs	4.27	11.3	1.38	4.28
LCFAs	29.4	77.4	27.2	85.2
SFAs	27.2	71.7	23.0	71.9
MUFAs	8.04	21.2	6.81	21.3
PUFAs	0.77	2.04	0.60	1.86

* FAs, fatty acids; BCFAs, branched-chain fatty acids; SCFAs, short-chain fatty acids; MCFAs, medium-chain fatty acids; LCFAs, long-chain fatty acids; SFAs, saturated fatty acids; MUFAs, monounsaturated fatty acids; PUFAs, polyunsaturated fatty acids.

### Analysis methodology

#### Chemicals

Sodium acetate trihydrate (pro analysis) was purchased from Merck KGaA (Darmstadt, Germany). Sulphosalicylic acid (>99%), chloroform (>99%), methanol (>99%), *iso*-octane (>99%) were purchased from VWR Chemicals BDH (Radnor, PA, USA). 2-Propanol (>99%), hexane (>99%), sodium methoxide (>95%) and glacial acetic acid (>90%) were purchased from Sigma-Aldrich (Auckland, New Zealand).

The Carboxen/PDMS solid-phase microextraction fibers, the internal standard mix (containing acetic acid-*d*_4_, propionic acid-*d*_3_, *iso*-butyric acid-*d*_7_, butyric acid-*d*_7_ and ^13^C-labeled isovaleric acid), and internal standard of C23:0 methyl ester were purchased from Supelco (Bellefonte, PA, USA). Internal standard for lipidomics (16:0 d31–18:1-PE) was purchased from Avanti (Polar Lipids, Inc., Alabaster, AL, USA).

#### Fatty acid analysis in milk

FAs (C2:0–C24:0) in milk samples were analyzed as follows: Cow or sheep milk powders (12.5 and 15.0 g, respectively) were reconstituted in 90 mL MilliQ water. Reconstituted milk (2.0 mL) was mixed with 2-propanol (4.0 mL), and hexane (3.0 mL) was then added. After shaking vigorously, the hexane layer was transferred to a new tube and evaporated to dryness. The residue was re-suspended in hexane (4.0 mL). Sodium methoxide in methanol (0.5 M, 100 μL) was added and the sample was vortexed for 5 s, repeating 4–5 times over 10 min. Glacial acetic acid (5 μL) was added to neutralize the sodium methoxide. The sample was dried by adding anhydrous calcium chloride to ~1 cm depth, and was then incubated at room temperature for 60 min. The top layer was collected and transferred to 1.5 mL GC vials and stored at 4 °C until analysis.

Fatty acid analysis was performed using a Shimadzu GC2030 gas chromatograph (GC) equipped with a flame ionization detector (FID). Fatty acids were separated on a Restek RTX 2,330 column (100 m × 0.22 mm ID × 0.2 μm film thickness; Restek Corporation, Bellefonte, PA, USA). The column oven was held at 75 °C for 5 min and was then increased to 175 °C at a rate of 15 °C/min and held for 17 min. Thereafter the temperature was increased to 250 °C at a rate of 5 °C/min and held for 6 min. The carrier gas was hydrogen with a linear velocity of 50 cm/s. The injection volume was 1 μL, with a split ratio of 60:1. The injector temperature was 260 °C and the detector temperature was 265 °C.

FAs in milk samples were reported as mg/mL and as a percentage of total FAs after peak areas were corrected for detector response using theoretical FID response factors. The equations for generating the response and conversion factors to quantify individual fatty acids from the FAMEs were obtained from American Oil Chemists' Society (AOCS Ce 1f-96, Ce 1h-05, and Ce 1i-07).

#### Lipidomic analysis in milk and plasma samples

Lipidomic analysis of the reconstituted milk, or of plasma, was conducted using the following method: Reconstituted milk or plasma (100 μL) was mixed with 800 μL of pre-chilled extraction solvent (CHCl_3_:MeOH = 1:1, v/v) and stored at -20°C for 60 min. MilliQ water (400 μL) was added, and the samples were centrifuged at 13,663×g for 10 min at 4 °C. The lower, organic layer (300 μL) was transferred to a centrifuge tube and evaporated to dryness under a stream of nitrogen at room temperature. Lipid residues were reconstituted in 300 μL of modified Folch solution (CHCl_3_:MeOH: H_2_O = 66:33:1, v/v/v, containing 0.01% w/v 16:0 d31-18:1-PE internal standard) and centrifuged (13,663×g, 4 °C, 10 min). Supernatant (20 μL) from each sample were pooled to form a quality control (QC). The remaining supernatant was used for lipid liquid chromatography-mass spectrometry (LC-MS) analysis. LC-MS analysis of lipids, raw data processing, lipid annotation and lipid identification were performed using a previously published method (26). Lipidomic analysis was performed using a Thermo UHPLC-MS system (Thermo Fisher Scientific, Waltham, MA, USA) comprising a Q-Exactive OrbitrapTM mass spectrometer with electrospray ionization. Samples were cooled in the auto-sampler at 4 °C and 2 μL of extract was injected onto a Waters Acquity CSH-C18 column (100 × 2.1 mm ID; 1.7 μm particle size) at 65 °C and eluted over a 17 min gradient with a flow rate of 600 μL/min. Mobile phase A was a mixture of isopropanol:acetonitrile (90:10, v/v with 0.1% formic acid and 10 mM ammonium formate) and mobile phase B was a mixture of acetonitrile:water (60:40, v/v with 0.1% formic acid and 10 mM ammonium formate). The gradient elution program was as follows: 0–2 (15–30% A), 2–2.5 (30–48% A), 2.5–11 (48–82% A), 11–11.5 (82–99% A), 11.5–14 (held at 99% A), 14–14.1 (99–15% A), and 14.1–17 min (held at 15% A).

Both full and data dependent MS^2^ (ddMS^2^) scans were collected in profile data acquisition mode. For full scan mode, a mass resolution setting of 35,000 was set to record a mass range of *m*/*z* 200–2,000 with a maximum trap fill time of 250 ms. In ddMS^2^, MS^2^ measurements are activated when a set peak intensity threshold is achieved. For ddMS^2^ scan mode, the same mass resolution setting was maintained with a maximum trap fill time of 120 ms. The isolation window of selected MS^1^ scans was ±1.5 *m*/*z* with a normalized collision energy of 30 eV. Samples were run in both positive and negative ionization modes separately. Positive ion mode parameters were as follows: spray voltage, 4.0 kV; capillary temperature, 275 °C; capillary voltage, 90 V; tube lens 120 V. Negative ion mode parameters were as follows: spray voltage, -2.5 kV; capillary temperature, 275 °C; capillary voltage, -90 V; tube lens, -100 V. The nitrogen source gas desolvation settings were the same for both modes (arbitrary units): sheath gas, 40; auxiliary gas, 10; sweep gas, 5. The Xcalibur software package provided by the manufacturer was used to create these settings.

The sequence of runs comprised blanks (containing no milk or plasma extract), QCs and samples in that order. To verify and maintain data quality, the QC sample was injected once every 10 samples. Retention time, signal intensity, and mass error of the internal standard were constantly monitored during the runs. For every 10 samples, one sample was selected at random for data dependent MS^2^ (ddMS^2^). Fragmentation data on 4 samples in total per ionization mode (positive and negative) were used for identification of lipid ions/classes.

Identification of lipids by LipidSearch^TM^ was based on comparison of the ddMS^2^ data obtained from select samples with *in silico* calculations/database. To ensure correct identification of lipid species by the LipidSearch^TM^ software, one lipid species from each class was manually identified according to published method (26).

#### Fatty acid analysis in plasma

SCFAs and MCFAs in plasma were analyzed as follows: Plasma (1.0 mL) was mixed with 20% sulphosalicylic acid (100 μL) and 100 μL internal standard mix. Samples were frozen overnight and were then centrifuged at 22,000×g for 45 min at 4 °C. Fatty acids were extracted from 300 μL of the supernatant using a Carboxen/PDMS solid-phase microextraction fiber for 5 min at room temperature. The fiber was then thermally desorbed at 280 °C for 5 min.

SCFAs and MCFAs were analyzed by gas chromatography-mass spectrometry (GC-MS) using a Shimadzu QP2010 instrument running in selected ion monitoring (SIM) mode on a Supelco Nukol column (30 m × 0.25 mm ID × 0.25 μm film thickness). The column oven was held at 80 °C for 1 min and was then increased to 200 °C at a rate of 75 °C/min and held there for 7 min. The carrier gas was helium with a linear velocity of 40 cm/s. The injection was thermally desorbing the fiber with a split ratio of 20:1, and the injector temperature was 280 °C. Ions were detected in SIM mode with an ion source temperature of 200 °C. The detector voltage was initially 1.3 kV, and was increased to 1.6 kV after 3.52 min. The *m*/*z* were monitored and used for quantitation.

LCFAs in plasma were analyzed as follows: Plasma (800 μL) was mixed with 1.0 mL internal standard (C23:0 methyl ester) and 8.0 mL chloroform:methanol 1:1 and was shaken for 10 min. Samples were then centrifuged at 2,000×g for 5 min at room temperature and the supernatant transferred to new tubes. Chloroform (4.0 mL) and MilliQ water (2.4 mL) were added, and samples were centrifuged at 2,000×g for 5 min at room temperature after which the upper aqueous layer was discarded. The solvent was evaporated from the chloroform phase at 40 °C under a stream of nitrogen. Methanolic NaOH solution (0.5 M, 1.5 mL) was added, and the samples were heated at 80 °C for 10 min. The samples were then cooled, mixed with methanolic BF_3_ solution (2.0 mL), and heated at 80 °C for 30 min. The samples were then cooled again, mixed with *iso*-octane (2.0 mL) for 1 min, then mixed with a saturated NaCl solution (5 mL) and finally centrifuged at 1,500×g for 5 min at room temperature. The *iso*-octane layer was transferred to glass vials and the remaining aqueous phase was extracted again using a further 1.0 mL of *iso*-octane. The combined *iso*-octane phases were evaporated to dryness at 40 °C under a stream of nitrogen. The residue was reconstituted in *iso*-octane (100 μL).

LCFAs were analyzed using the same equipment and column as the FA analysis in milk samples described in section 2.5.2. The column oven was held at 175 °C for 20 min and was then increased to 220 °C at a rate of 10 °C/min and held for 12 min. Thereafter the temperature was increased to 260 °C at a rate of 20 °C/min, and finally to 275 °C at a rate of 1 °C/min. The carrier gas was hydrogen with a linear velocity of 50 cm/s. The injection volume was 1 μL, with a split ratio of 30:1. The injector temperature was 300 °C and the detector temperature was 300 °C.

FA in plasma are reported as μg/mL. Quantitation of SCFAs and MCFAs was based on a 5-point calibration curve using the calibration curve function of the GCMS Solutions software (Shimadzu, Japan). The concentration in μg/mL plasma was calculated directly by GCMS Solutions and exported to MS-Excel for data analysis. LCFAs were quantitated after peak areas were corrected for detector response using theoretical FID response factors. The equations for generating the response and conversion factors to quantify individual fatty acids from the FAMEs were obtained from American Oil Chemists' Society (AOCS Ce 1f-96, Ce 1h-05, and Ce 1i-07). The corrected areas were multiplied by the internal standard (IS) ratio (IS concentration/IS area) to give μg FAME/sample. This value was divided by the sample volume (0.8 mL) to give μg/mL, this was then converted to μg FA/mL plasma by the ratio of the molecular weights of the fatty acid/FAME.

### Statistical analysis

A sample size of 30 was calculated for the primary outcome as described elsewhere (15). In brief, to detect a 20% difference in peak amino acid concentrations (608 ± 198 μmol/L^−1^) (27) with a power of 90% using α 0.05. Statistical analyses were performed with SPSS version 25 (SPSS, IBM Corporation, Armonk, NY, USA). Continuous data are presented as mean ± SEM. Study outcomes were analyzed on a per protocol basis. Incremental area under the curve (iAUC) was calculated using the trapezoidal method, correcting for baseline concentrations. Outliers in fatty acid and lipid data were identified as ≤ Q3 + 3IQR. Multiple imputation was used for values missing completely at random, as the mean of 5 iterations. Values lower than the limit of quantification were imputed at 50% of the limit of detection. Continuous variables were analyzed using parametric tests.

Single factor comparisons, such as iAUC, were made using Student's paired *t*-test with the null hypothesis that there is no difference between the test drink treatments. The null hypothesis was rejected, and the difference between treatments statistically significant, if *P* < α and *t* > *t*_crit_. All outcomes with multiple factors were analyzed by repeated factor generalized linear models with milk and time compared within-subject and adjusted for multiple comparisons using a Sidak Holm adjustment. The Huynh-Feldt correction was used where Mauchly's sphericity test failed. Alpha was set at *P* < 0.05 for all tests.

Heat maps were created using R software version 2.15.2 (28) with gplots (heatmap.2), RColorBrewer and colorRamps packages (R Development Core Team).

## Results

### Demographics

Thirty females (aged 24.4 ± 1.1 years, BMI 23.3 ± 1.1 kg/m^2^) completed the study. All subjects had lipid profile values within a healthy range (Table 3). All subjects indicated they “avoided drinking milk” (as a binary classification to determine eligibility). Based on lactase genotyping, 80% of the participants showed lactase non-persistence (LNP; i.e., CC13910 and GG22018) and only 20% exhibited lactase persistence (LP; i.e., CT13910 or TT13910 and GA22018 or AA22018).

**Table 3:** Anthropometric and biochemical characteristics of the participants at baseline.

**Measures**	**Mean**	**SEM**
Total cholesterol, mmol/L	4.35	0.11
Total triglycerides, mmol/L	0.95	0.04
HDL-C, mmol/L	1.54	0.05
LDL-C, mmol/L	2.60	0.11

^*^HDL-C, high-density lipoprotein cholesterol; LDL-C, low-density lipoprotein cholesterol.

### Postprandial lipemic response

**Figure 1:**
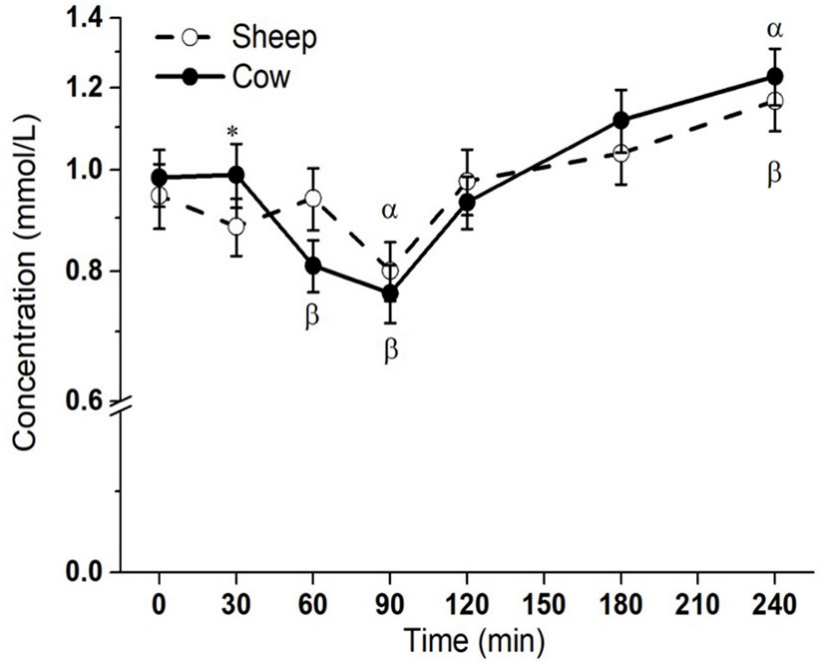
Postprandial changes in plasma total triglycerides after SM and CM ingestion. Values presented as means ± SEM; ^*^p < 0.05, ^**^p < 0.01, ^***^p < 0.001 denoted statistical significance (interaction time × milk) between sheep () and cow () milk; *α* and *β* denote significant changes (*p* < 0.05) from baseline after sheep and cow milk ingestion, respectively (Sidak corrected post hocs). SM, sheep milk; CM, cow milk.

Postprandial lipemic responses, or total blood triglyceride responses, were different between SM and CM treatments (interaction milk × time *p* = 0.003; Figure 1). Both SM and CM resulted in a TG decrease from baseline to 90 min, followed by an increase from this point to 240 min, at which point TG concentrations were elevated above baseline. Although TG concentrations were higher with CM at 30 min than sheep milk (*p* = 0.048), the overall post-meal response was similar between the milks, despite the difference in total fat intake.

### Lipidomic response

A total of 131 lipid species were detected in plasma following milk ingestion. The number of lipid classes detected in plasma (e.g., TGs, DGs, PC, PE, PI, PS, SphM, Cer, ZyE, etc.) was greater than in the test drinks themselves. In contrast, the number of TG species in plasma was only about 10% of the species found in the test drinks (37 in plasma compared to 362 in the test drinks).

**Figure 2:**
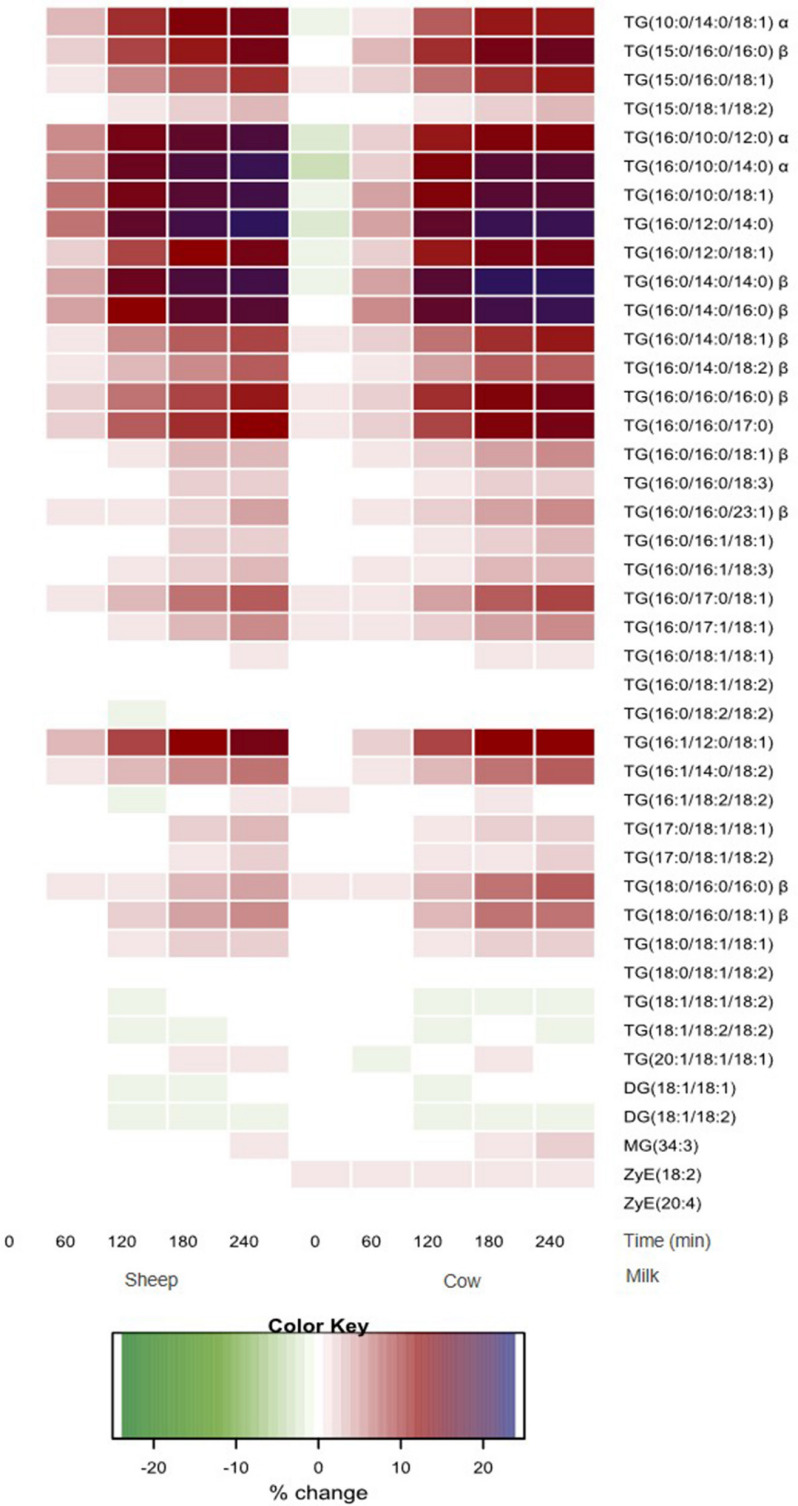
Heatmap of postprandial changes in individual TG, DG, MG and ZyE species. Values are presented as mean log fold% changes relative to concentrations at fasting SM (0 min); white represents a 0% change from SM baseline; red represents a 12.5% increase; blue represents and 25% increase; green represents a decrease; *α* denoted interaction time × milk with postprandial SM abundance greater than CM (*p* < 0.05), while *β* denoted postprandial CM abundance is greater than SM (*p* < 0.05). SM, sheep milk; CM, cow milk; TG, triglyceride; DG, diglyceride; MG, monoglyceride; ZyE, Zymosteryl ester.

The relative abundance of each lipid is presented as the log fold % change relative to the SM baseline concentrations to illustrate the pattern of response across all lipid species over time. As most differences dependent on milk type were in the meal-derived lipids, TGs, DGs, MGs, and ZyE are presented in Figure 2. Other lipid classes such as PLs, SphMs, LPCs, cholesteryl esters (ChE), and Cer are presented in Supplementary Figure 1. Most time effects were observed for the TG species. All TG species increased in plasma after milk ingestion (*p* < 0.05 main time effect), except for TG (18:0/18:1/18:2) (*p* > 0.05). In general, saturated TGs showed the greatest increase relative to the baseline SM concentrations, followed by the monosaturated TGs, while the polyunsaturated TGs changed the least. Changes in three species containing C10:0 [namely TG(10:0/14:0/18:1), TG(16:0/10:0/12:0) and TG(16:0/10:0/14:0), interaction time × milk *p* < 0.05] were greater after SM ingestion than CM, while 10 species with LCFAs [e.g., TG(15:0/16:0/16:0), TG(16:0/14:0/14:0), TG(16:0/14:0/16:0), TG(16:0/16:0/16), and TG(18:0/16:0/16:0), interaction time × milk *p* < 0.05; Figure 2] increased more in abundance postprandially with CM compared to SM. Similarly, the iAUC for TG(10:0/14:0/18:1), TG(16:0/10:0/12:0), and TG(16:0/10:0/14:0) in circulation was higher for SM than CM (*p* < 0.05; Supplementary Table 2). In contrast, the iAUC for TG(15:0/16:0/16:0), TG(16:0/14:0/14:0), TG(16:0/14:0/16:0), TG(16:0/16:0/16:0), and TG(18:0/16:0/16:0) in circulation was higher for CM than SM (*p* < 0.01; Supplementary Table 2).

Of the other lipid classes, only two were significantly different in circulation following ingestion of the milks. SphM(d34:1) was more abundant 1 h after SM ingestion (interaction time × milk *p* = 0.043; Supplementary Figure 1). PC(16:0/20:4) increased more with higher abundance with CM at 3 h (interaction time × milk *p* = 0.038; Supplementary Figure 1). However, the iAUC for the PL, SphM or ChE species were not significantly different between two milks (*p* > 0.05; Supplementary Table 2).

### Fatty acid response

**Figure 3:**
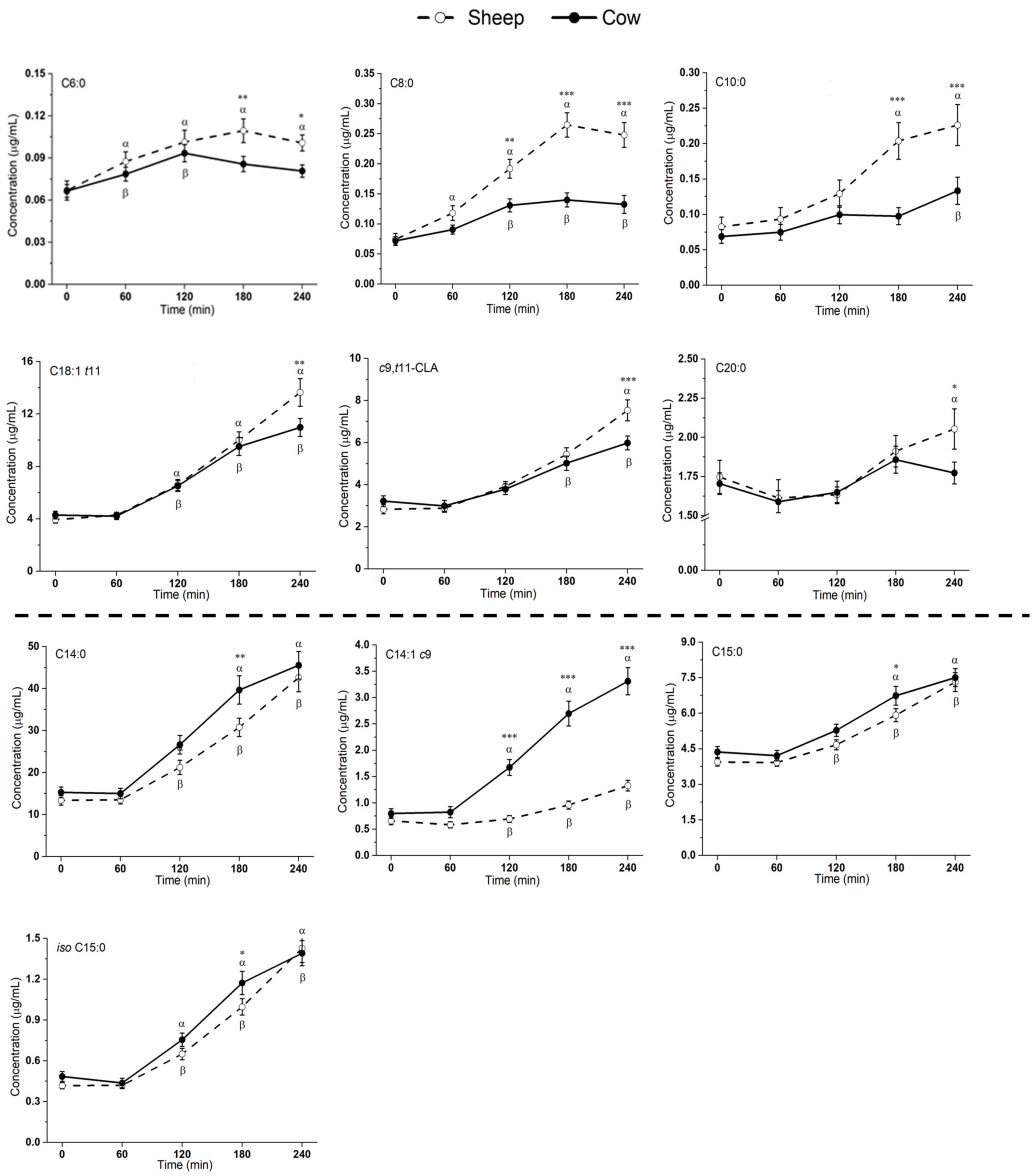
Postprandial changes in fatty acids that differed significantly in plasma following ingestion of SM and CM. Values presented as means ± SEM; *p < 0.05, **p < 0.01, ***p < 0.001 denoted statistical significance (interaction time × milk) between sheep () and cow () milk; *α* and *β* denote significant changes (*p* < 0.05) from baseline after sheep and cow milk ingestion, respectively (Sidak corrected post hocs). SM, sheep milk; CM, cow milk.

Most measured fatty acids (FAs) were responsive to milk ingestion (*p* < 0.05; Figure 3 and Supplementary Figure 2), aside from PUFAs, e.g., C18:2n6, C18:3n3, C20:3n6, C20:4n6, C20:5n3, C22:5, and C22:6n3 (*p* > 0.05) and FAs with low abundance, e.g., C5:0 and C7:0 (*p* > 0.05).

Plasma levels of C6:0, C8:0, and C10:0 increased to a greater extent following SM ingestion than CM ingestion (interaction time × milk *p* < 0.05; Figure 3). The trend was the same for the following LCFAs: C18:1 *t*11, *c*9, *t*11-CLA, and C20:0 (*p* < 0.05). These fatty acids also showed a greater iAUC with SM than CM, aside from C20:0 (*p* = 0.932; Table 4). Additionally, the iAUC for C20:1 *c*11 was greater with SM (*p* < 0.01; Table 4), despite no interaction of milk × time (*p* > 0.05) and is likely due to the relative low abundance of this FA, combined with differences observed at baseline, which impacts the iAUC calculations. *Iso* C5:0, *anteiso* C15:0, and *iso* C16:0 were more abundant in circulation after SM than CM, although this was not dependent on time (interaction of milk × time *p* > 0.05; Supplementary Figure 2); as this difference was not observed in the iAUC (*p* > 0.05; Table 4), this main effect is more likely due to the differences in baseline concentrations.

**Table 4:** Fatty acid iAUC (μg·min/mL) in blood plasma following sheep and cow milk ingestion.

**FA**	**Sheep milk**	**Cow milk**	***p-*value**
**SCFAs**
C2:0	158.7 ± 51.1	180.6 ± 77.2	0.713
C3:0	10.4 ± 3.01	10.2 ± 3.27	0.956
C4:0	13.2 ± 3.13	13.9 ± 3.22	0.770
C5:0	0.16 ± 0.40	0.15 ± 0.48	0.984
C6:0	6.89 ± 1.09	4.02 ± 0.71	< 0.05
**MCFAs**
C7:0	−1.82 ± 1.90	−0.57 ± 0.78	0.546
C8:0	26.4 ± 1.61	10.6 ± 1.01	< 0.001
C10:0	15.0 ± 2.01	5.80 ± 1.40	< 0.001
**LCFAs**
C14:0	2,398 ± 242	2,955 ± 308	0.068
C15:0	278.3 ± 31.4	268.4 ± 33.4	0.782
C16:0	2,529 ± 1,541	4,022 ± 1,736	0.425
C17:0	138.2 ± 31.0	95.1 ± 20.5	0.203
C18:0	1,639 ± 430	1,284 ± 483	0.510
C20:0	22.4 ± 40.4	18.1 ± 37.4	0.932
**BCFAs**
*iso* C5:0	3.31 ± 1.52	4.58 ± 1.02	0.439
*iso* C15:0	51.5 ± 15.5	52.7 ± 15.8	0.956
*anteiso* C15:0	177.4 ± 28.8	170.4 ± 59.8	0.914
*iso* C16:0	53.5 ± 22.1	67.2 ± 25.1	0.677
*iso* C17:0	61.4 ± 33.3	−12.8 ± 45.5	0.120
**MUFAs**
C14:1 *c*9	1.83 ± 18.8	218.5 ± 33.0	< 0.001
C16:1 *c*9	−296.3 ± 192.6	−199.9 ± 228.4	0.630
C17:1 *c*9	17.9 ± 19.5	27.2 ± 19.7	0.717
C18:1 *c*9	−5,934 ± 1,895	−6,219 ± 2,054	0.897
C18:1 *c*11	−254.5 ± 112.6	−357.6 ± 129.5	0.440
C18:1 *t*9	63.8 ± 15.6	21.9 ± 20.0	0.091
C18:1 *t*11	845.1 ± 66.7	630.1 ± 56.0	< 0.01
C20:1 *c*11	−4.58 ± 50.3	−169.7 ± 31.3	< 0.01
**PUFAs**
*c*9, *t*11–CLA	374.3 ± 34.1	198.0 ± 27.1	< 0.001
C18:2n6	−3,648 ± 2,510	−6,635 ± 3,095	0.497
C18:3n3	−107.1 ± 66.2	−280.0 ± 106.3	0.109
C18:3n6	109.2 ± 84.8	−54.6 ± 64.4	0.145
C20:3n6	125.3 ± 90.4	302.2 ± 301.0	0.594
C20:4n6	390.0 ± 444.7	431.9 ± 577.3	0.958
C20:5n3	57.6 ± 63.9	38.0 ± 63.0	0.827
C22:5	59.0 ± 31.5	13.3 ± 46.8	0.445
C22:6n3	171.0 ± 134.9	−7.59 ± 180.6	0.469

^*^iAUC, incremental area under the curve; FAs, fatty acids; BCFAs, branched-chain fatty acids; SCFAs, short-chain fatty acids; MCFAs, medium-chain fatty acids; LCFAs, long-chain fatty acids; SFAs, saturated fatty acids; MUFAs, monounsaturated fatty acids; PUFAs, polyunsaturated fatty acids.

The increase in plasma concentrations of several FAs such as C14:0, C14:1 *c*9, C15:0, and *iso* C15:0 (^*^p < 0.05 interaction time × milk) was greater after CM digestion compared to SM. Of these, only C14:1 *c*9 showed a similarly higher iAUC with CM (*p* < 0.001; Table 4). Greater abundances of C16:0 and C18:3n6 were detected in circulation after CM than SM (*p* < 0.05 main milk effect; Supplementary Figure 2). This is likely due to the baseline differences in these FAs, leading to no difference in iAUC.

**Figure 4:**
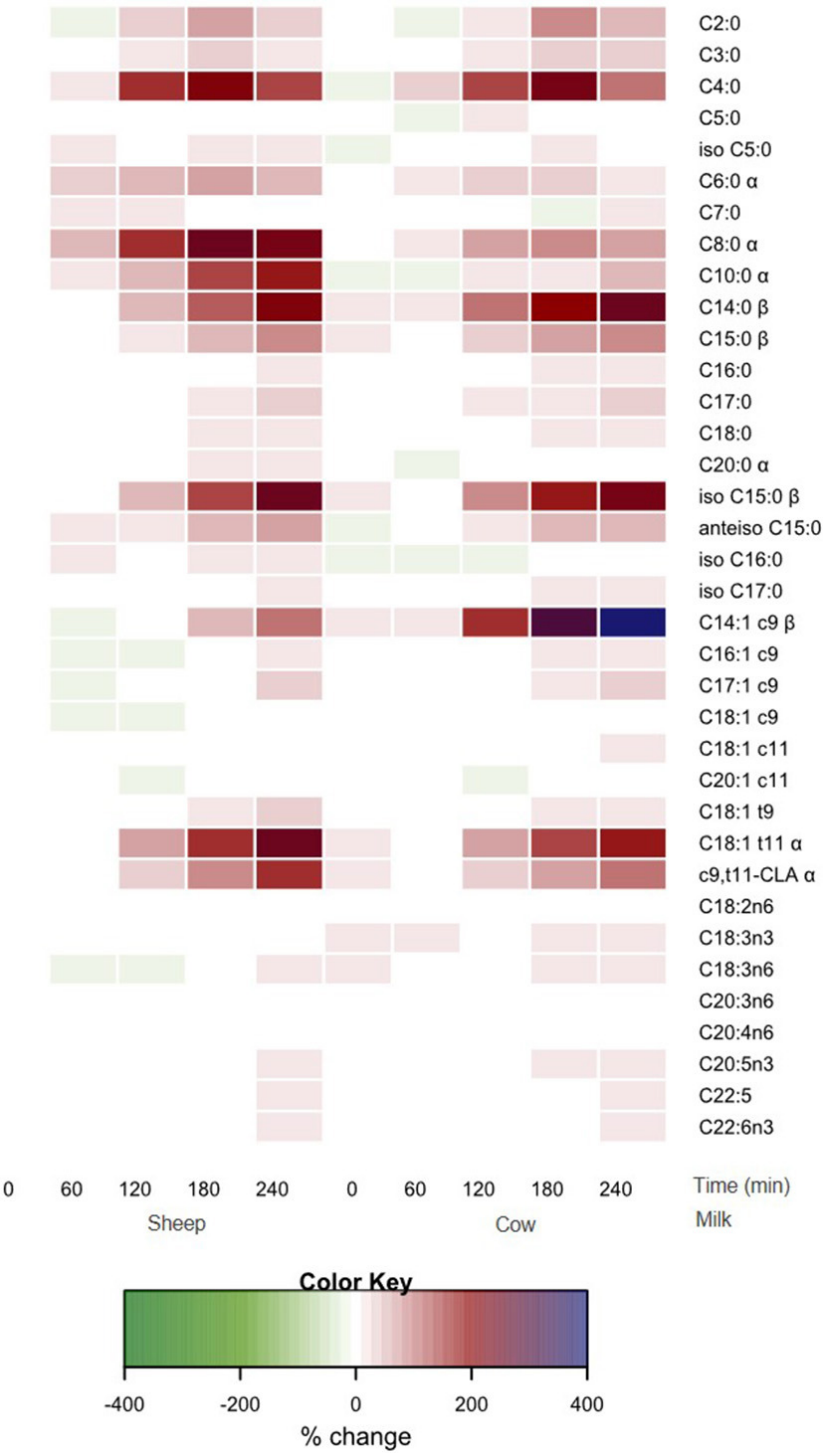
Heat map of postprandial changes in individual plasma fatty acid after SM and CM ingestion. Values are presented as mean log fold % changes relative to concentrations at fasting SM (0 min); white represents a 0% change from SM baseline; red represents a 50% increase; blue represents and 100% increase; green represents a decrease; *α* denoted interaction time × milk with postprandial SM abundance greater than CM (*p* < 0.05), while *β* denoted postprandial CM abundance is greater than SM (*p* < 0.05). SM, sheep milk; CM, cow milk.

All individual fatty acid responses are shown as a heatmap (Figure 4) displaying the percentage change from SM fasting concentrations (0 min). SCFAs and MCFAs concentrations changed between −10 and +257% from SM fasting concentrations, while LCFAs and PUFAs changed <40% except for C14:1 *c*9, C18:1 *t*9, C18:1 *t*11, and *c*9, *t*11-CLA. These fatty acid concentrations changed between −11 and 402%.

## Discussion

The overall lipemic response, or total blood TG response, was very similar for both milks, despite the greater total fat content in SM. Graded lipemic effects (TG iAUC) have been demonstrated for ingested quantities of 15 vs. 30 g of fat, but this relationship does not continue for intakes >30 g (29). However, in this study, despite SM containing a ~1.6-fold higher fat content than CM (33.4 vs. 21.3 g), the similar overall lipemia is likely due in part to the compositional differences in lipid species, particularly the high abundance of MCFAs in SM. Indeed, the only difference in total TG concentrations between SM and CM was found at 30 min, earlier than the expected appearance of meal-derived lipids in circulation from 120 min, and at total TG concentrations that were similar to fasting. There is however a possible impact of the higher protein content in SM on the similar lipemia from 2 h onwards; protein content is known to suppress postprandial lipemia (15, 16, 30). The digestive differences between casein and whey have similarly been shown to impact lipemia (31, 32), and structural differences in sheep vs. cow casein and whey properties could be considered as a possible mechanism to explain the similar lipemic responses (33).

As shown in the compositional analysis of the reconstituted milk samples, SM has a higher proportion of SCFAs and MCFAs (C4:0–C10:0) (Table 2). SM containing greater concentrations of MCFAs results in a greater proportion of TGs containing C10:0. In the current study greater abundances of TG(10:0/14:0/18:1) were measured after SM ingestion. The positional distribution of these FAs in the TG moiety also differs between milk types, as C10:0 in the *sn*-1/-3 positions are more abundant in SM compared to CM according to our previous *in vitro* work (20). Whilst the method used in the current study only provides tentative stereospecific numbering (*sn*-) of fatty acids in the TG backbone, the postprandial plasma response demonstrated greater abundances of C10:0-containing TGs, including TG(10:0/14:0/18:1), TG(16:0/10:0/12:0), and TG(16:0/10:0/14:0), after SM ingestion. However, many other differences in FAs between milk types were not evident in the TG fraction. For the SCFAs and MCFAs, this may be reflective of the rapid absorption and subsequent mitochondrial β-oxidation in the liver (34, 35). This source of energy available for β-oxidation may be biologically beneficial for those with higher energy requirements such as infants or athletes (36, 37). It is notable that human breast milk is a rich source of MCTs (~15% of total lipids) that are necessary for the growing human infant because of their ease of digestion and rapid availability as an energetic substrate (38).

CM has a greater abundance of some LCFAs (C14:0–C18:0), and there were some notable differences in the LCFA species between the milks (Table 2). It has previously been reported that TGs containing C18:1 in the *sn*-1/-2 position are more abundant in SM (20). In general, lipid species that were present in greater abundance in the respective milks showed a similar difference in circulation. For example, CM contained ~4-fold higher abundance and 5-fold higher proportion of C14:1 c9 (myristoleic acid) than SM (Table 2). This was reflected correspondingly by ~2.5-fold higher concentrations at 120–240 min in circulation (Figure 3) and ~120-fold higher iAUC after CM ingestion compared to SM (Table 4). however compositional differences in SCFA and LCFAs are less apparent in the circulating plasma in the postprandial period. Further analysis is required to determine whether this finding is a consequence of differences in metabolic fate, altered flux into lipids such as ceramides or altered digestibility.

The lipidomic analysis performed in this study also included other major classes of lipid species, including PLs and SphM, which have important signaling and regulatory roles, particularly within cellular membranes (39). Milk PLs and SphM are of particular interest for their possible beneficial impacts on the gut health and brain development of infants (40). Previous postprandial studies have shown these lipid species undergo significant reconfiguration and metabolism once absorbed (41). Therefore, circulating concentrations and species of lipids are only partially reflective of dietary intake. In the current study we identified a 37-fold higher abundance of SphM(d34:1) in the SM, compared to the CM (Supplementary Table 1). There was correspondingly a greater abundance of this SphM in circulation following SM ingestion, although the difference at any time point was <1-fold. Notably, two further SphM species were more abundant in SM [SphM(d32:0) and SphM(d36:4) (144-fold and 174-fold higher than CM, respectively; Supplementary Table 1)], yet there were no differences in the measured levels of these SphM species in circulation. Therefore, as the dairy sector continues to explore possible functionality of these minor lipids for clinical application, analysis is required not only of the content in the food/beverage product, but also their metabolic fate as product lipid content may not match that of the circulatory response.

*Trans* fatty acids (TFAs) are a class of unsaturated fatty acids in which one or more double bonds are present in the so-called *trans* configuration. The most common origin of TFAs in the Western diet is industrially generated (predominately C18:1*t*9), produced with the partial hydrogenation of vegetable oils (42). There is extensive evidence that excessive intake of industrially generated TFAs increase the risk of cardiovascular disease (CVD) (43, 44). However, ruminant sourced TFAs differ, with C18:1 *t*11 (*trans*-vaccenic acid) being the most abundant in milk (45). Ruminant TFAs may have minimal impact on cardiometabolic risk (46, 47), with no convincing association between ruminant TFAs and CVD (48, 49). In the current study, a 1.26-fold higher abundance of C18:1 *t*11 (*trans*-vaccenic acid) was found in the SM (Table 2). This in turn was measurable in the plasma, with 1.24-fold higher (*p* < 0.01) concentration at 240 min (Figure 3) and 1.34-fold higher iAUC after SM ingestion (Table 4). Whether the differences in TFA composition between SM and CM is biologically important and can impact on cardiovascular risk is not known.

Conjugated linoleic acid (CLA) is a term referring to a family containing many different FAs with 18 carbons and two conjugated double bonds (50). The most abundant CLA in CM is *c*9, *t*11-CLA (rumenic acid) (11). Rumenic acid, and some of the other acids in the CLA family, contain *trans* double bonds and are therefore also TFAs. CLA may be beneficial in a wide range of health conditions, including atherosclerosis, diabetes, obesity and as a beneficial modulator of the immune system (51). In the current study, a 1.19-fold higher abundance of *c*9, *t*11-CLA was found in the SM (Table 2). This in turn was measurable in the plasma, with 1.26-fold higher (*p* < 0.01) concentration at 240 min (Figure 3) and 1.89-fold higher iAUC after SM ingestion (Table 4). Whether the differences in *c*9, *t*11-CLA concentration between SM and CM is biologically functional is not known.

Our study included a small cohort of healthy women aged 20–40 years, and the results may not be generalizable to men, people of a different age or those with different metabolic health status. The subjects were also those who self-reported dairy avoidance, with the possibility their digestion may be altered relative to the wider community. Analysis was performed on a fixed volume of milk, made from reconstituted powder. The benefit of MCFAs provided by SM needs to be further studied. Given the inherent differences in lipid composition, the sheep milk in this study resulted in a greater quantity of lipids ingested. To fully elucidate the differences in lipid digestibility, metabolism and lipidomic response, studies are required that compare equal quantities of total lipids. However, such a study is not reflective of how foods are consumed (typically based on serving size).

This study provides a highly comprehensive determination of the lipidome with 415 species detected in SM and CM, and 131 species in blood plasma. The importance of lipidomic analysis, beyond the insights that are gained with measurement of FA species alone is detailed data on the relative distribution of FAs into the differing biological active lipid compartments including TGs, PLs and SphM. As highlighted in this study, there is extensive SM metabolism, with the composition of the ingested beverage only partially relating to the circulating SM lipidome.

In conclusion, the ingestion of SM and CM results in different circulatory lipidome responses when compared “portion-for-portion.” Although providing a ~1.6-fold higher fat content, SM does not result in a greater circulating increase of total postprandial triglyceride compared to CM. SM results in a greater circulating increase in three TG species containing C10:0, SCFAs and MCFAs (i.e., C6:0, C8:0, and C10:0) and several long-chain fatty acids (LCFAs) (i.e., C18:1 *t*11, *c*9, *t*11-CLA, and C20:0) compared to CM. These differences are not only a result of compositional differences between SM and CM, but also attributed to the inherent differences in FA positioning and the lipid digestibility. The greater uptake of MCFAs may make SM a potential fast energy source for consumers seeking CM alternatives, especially for those with higher energy requirements such as infants or athletes. In addition, the greater availability and uptake of potentially biologically beneficial lipids including C18:1 *t*11 and *c*9, *t*11-CLA with SM warrant further investigation into the role of SM in human health.

## Data availability statement

The original contributions presented in the study are included in the article/Supplementary material, further inquiries can be directed to the corresponding author.

## Ethics statement

The study involving human participants was reviewed and approved by Central Health and Disability Ethics Committees, Ministry of Health, Ethics Department, 133 Molesworth Street Wellington 6145 New Zealand. The patients/participants provided their written informed consent to participate in this study.

## Author contributions

FT conducted research, analyzed data, performed statistical analyses, and wrote the first draft of the paper. LS designed research, managed the project, performed statistical analyses, and wrote the paper. AM designed and conducted research, performed statistical analyses, and wrote the paper. ASu analyzed lipids in milk and blood samples. MA analyzed fatty acids in milk and blood samples. ASh conducted research, analyzed data, and performed statistical analyses. DC-S designed the research and reviewed the paper. LD designed research, reviewed the paper, and had primary responsibility for the final content. All authors approved the final version of the manuscript before submission.
