# Supplementary material for: Postprandial lipemic response in dairy-avoiding females following an equal volume of sheep milk relative to cow milk: A randomized controlled trial
Source: Front Nutr. 2023 Jan 4;9:1029813. doi: 10.3389/fnut.2022.1029813 (PMC9846784; doi:10.3389/fnut.2022.1029813)
Supplement: Supplementary file 1 [file Data_Sheet_1.docx]

# Supplementary Table 1: Lipid composition of the sheep and cow milk test drinks^*^.

| **Lipids** | **Sheep milk** | | **Cow milk** | |
| --- | --- | --- | --- | --- |
|  | **1×10^9^ AU/mL** | **% total**  **TGs** | **1×10^9^ AU/mL** | **% total**  **TGs** |
| **TGs** |  |  |  |  |
| ECN<30 | 28.8 | 9.31 | 5.84 | 3.45 |
| ECN30 | 16.7 | 5.40 | 8.97 | 5.31 |
| ECN31 | 1.43 | 0.46 | 0.60 | 0.36 |
| ECN32 | 9.73 | 3.14 | 15.4 | 9.13 |
| ECN33 | 7.02 | 2.27 | 2.53 | 1.50 |
| ECN34 | 42.5 | 13.7 | 15.3 | 9.04 |
| ECN35 | 4.83 | 1.56 | 1.54 | 0.91 |
| ECN36 | 18.3 | 5.91 | 7.74 | 4.58 |
| ECN37 | 3.64 | 1.17 | 3.65 | 2.16 |
| ECN38 | 42.5 | 13.7 | 14.5 | 8.61 |
| ECN39 | 5.60 | 1.81 | 1.86 | 1.10 |
| ECN40 | 23.3 | 7.51 | 17.0 | 10.1 |
| ECN41 | 3.97 | 1.28 | 1.29 | 0.76 |
| ECN42 | 21.9 | 7.07 | 13.1 | 7.75 |
| ECN43 | 3.68 | 1.19 | 2.30 | 1.36 |
| ECN44 | 22.6 | 7.29 | 9.33 | 5.52 |
| ECN45 | 4.38 | 1.43 | 3.27 | 1.95 |
| ECN46 | 18.4 | 5.58 | 18.3 | 9.92 |
| ECN47 | 3.26 | 1.39 | 0.67 | 1.28 |
| ECN48 | 5.12 | 1.65 | 6.88 | 4.07 |
| ECN49 | 2.75 | 0.89 | 2.23 | 1.32 |
| ECN50 | 9.14 | 2.95 | 8.62 | 5.10 |
| ECN51 | 1.28 | 0.41 | 1.04 | 0.61 |
| ECN52 | 4.36 | 1.41 | 3.86 | 2.29 |
| ECN53 | 1.07 | 0.35 | 0.75 | 0.44 |
| ECN54 | 1.78 | 0.57 | 0.98 | 0.58 |
| ECN>54 | 5.30 | 0.56 | 3.39 | 0.85 |
| MCTs (CN≤36) | 92.7 | 41.8 | 45.3 | 34.3 |
| LCTs (CN>36) | 217.1 | 58.2 | 123.7 | 65.7 |
| SCTs (DB=0) | 110.7 | 35.7 | 68.3 | 40.4 |
| MUCTs (DB=1) | 100.4 | 32.4 | 57.5 | 34.0 |
| PUCTs (DB≥2) | 98.8 | 31.9 | 43.2 | 25.6 |
| **DGs** | **1×10^6^ AU/mL** | **% total**  **DGs** | **1×10^6^ AU/mL** | **% total**  **DGs** |
| DG(8:0/10:0) | 5.02 | 1.62 | 1.32 | 0.17 |
| DG(10:0/10:0) | 12.5 | 4.02 | 1.92 | 0.25 |
| DG(10:0/12:0) | 14.0 | 4.52 | 4.62 | 0.61 |
| DG(4:0/18:2) | 5.49 | 1.77 | 1.78 | 0.24 |
| DG(10:0/14:0) | 22.0 | 7.07 | 3.72 | 0.49 |
| DG(12:0/14:0) | 45.9 | 14.8 | 65.1 | 8.62 |
| DG(10:0/18:1) | 0.44 | 0.14 | 1.59 | 0.21 |
| DG(16:0/14:0) | 2.51 | 0.81 | 36.1 | 4.78 |
| DG(16:0/14:0) | 18.1 | 5.82 | 68.8 | 9.12 |
| DG(18:1/12:0) | 6.35 | 2.05 | 16.8 | 2.22 |
| DG(18:1/12:0) | 8.25 | 2.66 | 42.6 | 5.64 |
| DG(16:0/16:0) | 26.6 | 8.57 | 93.8 | 12.4 |
| DG(16:0/16:0) | 8.25 | 2.66 | 53.4 | 7.07 |
| DG(18:1/14:0) | 26.0 | 8.38 | 55.0 | 7.29 |
| DG(18:1/14:0) | 13.0 | 4.19 | 40.0 | 5.31 |
| DG(16:0/18:1) | 62.3 | 20.1 | 138.1 | 18.3 |
| DG(16:0/18:1) | 2.38 | 0.77 | 48.2 | 6.38 |
| DG(16:0/18:2) | 1.12 | 0.36 | 1.33 | 0.18 |
| DG(18:0/18:1) | 5.13 | 1.65 | 41.6 | 5.51 |
| DG(18:1/18:1) | 25.1 | 8.09 | 39.2 | 5.19 |
| **PC** | **1×10^6^ AU/mL** | **% total**  **PC** | **1×10^6^ AU/mL** | **% total**  **PC** |
| LPC(16:0) | 3.89 | 0.27 | 1.71 | 0.16 |
| PC(24:0) | 2.21 | 0.15 | 1.17 | 0.11 |
| PC(26:0) | 10.0 | 0.70 | 4.34 | 0.41 |
| PC(28:0) | 26.5 | 1.85 | 25.7 | 2.40 |
| PC(30:0) | 113.9 | 7.95 | 166.4 | 15.6 |
| PC(31:0) | 6.64 | 0.46 | 14.2 | 1.33 |
| PC(32:0) | 117.0 | 8.17 | 10.5 | 0.98 |
| PC(32:1) | 62.8 | 4.38 | 71.6 | 6.69 |
| PC(32:2) | 5.23 | 0.37 | 7.66 | 0.72 |
| PC(33:1) | 10.8 | 0.75 | 19.4 | 1.82 |
| PC(34:0) | 86.7 | 6.05 | 37.1 | 3.46 |
| PC(16:0/18:1) | 17.0 | 1.18 | 17.9 | 1.67 |
| PC(34:1) | 497.8 | 34.7 | 349.1 | 32.6 |
| PC(34:2) | 102.5 | 7.15 | 84.1 | 7.86 |
| PC(34:3) | 12.5 | 0.87 | 20.7 | 1.94 |
| PC(36:1) | 149.4 | 10.4 | 76.0 | 7.10 |
| PC(36:2) | 138.7 | 9.68 | 101.6 | 9.49 |
| PC(36:3) | 59.0 | 4.12 | 46.9 | 4.38 |
| PC(36:4) | 10.3 | 0.72 | 14.1 | 1.31 |
| **PE** | **1×10^6^ AU/mL** | **% total**  **PE** | **1×10^6^ AU/mL** | **% total**  **PE** |
| PE(16:0/18:1) | 1.00 | 2.46 | 21.3 | 24.3 |
| PE(18:0/18:1) | 8.17 | 20.1 | 27.0 | 30.9 |
| PE(18:1/18:1) | 4.90 | 12.1 | 21.4 | 24.5 |
| PE(18:1/18:2) | 26.5 | 65.3 | 17.8 | 20.3 |
| **SphM** | **1×10^6^ AU/mL** | **% total**  **SphM** | **1×10^6^ AU/mL** | **% total**  **SphM** |
| SphM(d32:0) | 2.44 | 0.25 | 0.02 | 0.01 |
| SphM(d32:1) | 30.5 | 3.18 | 23.6 | 13.5 |
| SphM(d33:1) | 0.29 | 0.03 | 0.30 | 0.17 |
| SphM(d34:0) | 1.10 | 0.11 | 13.8 | 7.85 |
| SphM(d34:1) | 74.7 | 77.9 | 19.7 | 11.2 |
| SphM(d36:4) | 32.8 | 3.42 | 0.19 | 0.11 |
| SphM(d39:1) | 12.1 | 1.26 | 67.7 | 38.6 |
| SphM(d41:1) | 129.1 | 13.5 | 44.5 | 25.4 |
| SphM(d42:1) | 3.79 | 0.40 | 5.43 | 3.10 |
| **Cer** | **1×10^6^ AU/mL** |  | **1×10^6^ AU/mL** |  |
| Cer(d34:0) | 10.7 | / | 11.6 | / |
|  | **1×10^6^ AU/mL** | **% total**  **PLs** | **1×10^6^ AU/mL** | **% total**  **PLs** |
| PC+LPC | 1433.0 | 58.7 | 1070.1 | 80.2 |
| PE | 40.6 | 1.67 | 87.6 | 6.57 |
| SphM | 959.3 | 39.4 | 175.2 | 13.1 |

^*^AU: arbitrary units; TGs: triglycerides; ECN (equivalent carbon number) =CN (total carbon number)-2×DB (double bond); MCTs: medium-chain triglycerides; LCTs: long-chain triglycerides; SCTs: saturated triglycerides; MUCTs: monounsaturated triglycerides; PUCTs: polyunsaturated triglycerides; PLs: phospholipids.

**Supplementary Table 2:** **Lipid iAUC (1×10^9^** **AU·min/mL) in blood plasma following sheep and cow milk ingestion.**

| **Lipid** | **Sheep milk** | **Cow milk** | ***p* value** |
| --- | --- | --- | --- |
| **TGs** | | | |
| TG(16:0/16:0/16:0) | 3.97±4.05 | 7.12±5.83 | <0.001 |
| TG(16:0/14:0/16:0) | 3.61±3.37 | 6.32±5.15 | <0.001 |
| TG(16:0/10:0/12:0) | 1.70±1.93 | 0.89±0.647 | <0.001 |
| TG(16:0/14:0/14:0) | 3.30±3.28 | 5.16±4.38 | <0.01 |
| TG(15:0/16:0/16:0) | 1.00±0.93 | 1.45±1.05 | <0.01 |
| TG(18:0/16:0/16:0) | 1.65±3.98 | 3.60±5.29 | <0.01 |
| TG(10:0/14:0/18:1). | 22.4± 2.87 | 1.32±1.15 | <0.01 |
| TG(16:0/10:0/14:0) | 2.39±2.89 | 1.57±1.22 | <0.05 |
| TG(16:0/12:0/18:1) | 5.60±7.18 | 7.57±6.62 | <0.05 |
| TG(16:0/16:0/17:0) | 1.15±1.02 | 0.88±0.10 | 0.068 |
| TG(16:0/14:0/18:1) | 9.94±11.5 | 13.0±12.1 | 0.069 |
| TG(16:0/10:0/18:1) | 4.68±5.67 | 3.51±2.95 | 0.075 |
| TG(16:0/17:0/18:1) | 12.8±1.47 | 17.0±1.86 | 0.086 |
| TG(16:1/12:0/18:1) | 35.1±4.72 | 2.68±2.44 | 0.132 |
| TG(16:0/16:0/18:1) | 9.16±1.49 | 12.1±12.45 | 0.146 |
| TG(18:0/16:0/18:1) | 3.82±5.90 | 5.04±6.97 | 0.191 |
| TG(16:1/14:0/18:2) | 1.63±2.23 | 2.05±2.36 | 0.204 |
| TG(16:0/16:0/23:1) | 2.78±0.49 | 3.57 ±0.53 | 0.281 |
| TG(18:1/18:1/18:2) | -1.04±13.3 | -2.64±9.79 | 0.348 |
| TG(16:0/16:0/18:3) | 4.23±10.8 | 2.99±7.23 | 0.355 |
| TG(16:0/16:1/18:3) | 11.8±2.29 | 9.41±1.60 | 0.397 |
| TG(16:1/18:2/18:2) | -0.13±2.88 | -0.46±3.38 | 0.465 |
| TG(17:0/18:1/18:1) | 4.93±1.32 | 6.34±1.44 | 0.475 |
| TG(16:0/17:1/18:1) | 13.3±2.41 | 1.12±1.64 | 0.483 |
| TG(18:1/18:2/18:2) | -0.88±7.19 | -1.62±7.54 | 0.484 |
| TG(16:0/14:0/18:2) | 6.25±8.93 | 7.03±6.70 | 0.497 |
| TG(15:0/16:0/18:1) | 21.1±2.64 | 2.34±2.38 | 0.521 |
| TG(15:0/18:1/18:2) | 5.71±1.50 | 0.45±1.34 | 0.538 |
| TG(16:0/18:1/18:2) | -2.81±44.3 | -4.86±3.29 | 0.715 |
| TG(20:1/18:1/18:1) | 0.64±0.44 | 0.39±0.54 | 0.722 |
| TG(17:0/18:1/18:2) | 0.25±1.24 | 0.32±1.47 | 0.732 |
| TG(16:0/16:1/18:1) | 9.00±1.97 | 8.20±1.34 | 0.742 |
| TG(16:0/18:1/18:1) | 2.50±42.8 | 4.21±32.6 | 0.752 |
| TG(18:0/18:1/18:2) | 0.78±15.4 | 0.23±9.04 | 0.763 |
| TG(16:0/12:0/14:0) | 2.96±2.99 | 3.07±2.62 | 0.768 |
| TG(16:0/18:2/18:2) | -2.78±24.8 | -1.99±22.2 | 0.814 |
| TG(18:0/18:1/18:1) | 1.33±3.96 | 1.48±4.83 | 0.815 |
| **DGs** | | | |
| DG(18:1/18:1) | -2.03±0.66 | -0.60±0.65 | 0.128 |
| DG(18:1/18:2) | -2.37±0.68 | -0.27±0.97 | 0.744 |
| **MGs** | | | |
| MG(34:3) | 0.99±0.64 | 0.88±0.62 | 0.911 |
| **ZyE** | | | |
| ZyE(18:2) | -0.20±0.79 | -0.11±1.25 | 0.527 |
| ZyE(20:4) | -0.02±0.70 | 0.02±0.88 | 0.851 |
| **PC** | | | |
| PC(34:3) | -0.62±17.6 | 3.92±18.4 | 0.080 |
| PC(34:2) | 43.9±28.3 | -22.7±34.8 | 0.142 |
| PC(35:2) | 0.75±8.62 | -0.89±9.63 | 0.209 |
| PC(38:4) | 9.77±63.6 | 0.27±60.4 | 0.284 |
| PC(16:0/20:4) | 0.01±0.12 | 0.19±0.14 | 0.331 |
| PC(16:1/18:1) | 1.46±16.6 | 2.74±24.5 | 0.668 |
| PC(38:5) | 3.38±23.5 | 1.95±30.3 | 0.711 |
| PC(16:0p/20:4) | 0.31±0.57 | 0.48±0.66 | 0.848 |
| PC(32:0) | 1.06±7.95 | 1.08±9.29 | 0.987 |
| PC(40:5) | 0.94±5.60 | -0.31±5.74 | 0.127 |
| PC(38:6) | 10.5±57.1 | -2.60±77.7 | 0.178 |
| PC(33:2) | 0.47±3.02 | -0.13±3.54 | 0.205 |
| PC(36:4e) | 2.15±12.7 | -0.16±13.1 | 0.210 |
| PC(38:3) | 1.57±11.7 | -0.07±14.0 | 0.375 |
| PC(32:1) | 1.71±7.15 | 0.73±9.76 | 0.421 |
| PC(36:2) | 24.0±167.2 | 4.82±170.6 | 0.425 |
| PC(34:2p) | 0.74±9.66 | -0.12±5.09 | 0.454 |
| PC(36:4) | 13.9±100.2 | 2.78±115.4 | 0.472 |
| PC(36:1) | 2.23±16.1 | 0.54±17.0 | 0.475 |
| PC(36:5) | 1.25±8.14 | 1.93±10.9 | 0.619 |
| PC(40:6) | 2.00±15.8 | 1.14±14.3 | 0.691 |
| PC(36:3) | 14.3±106.9 | 7.71±146.3 | 0.721 |
| PC(18:3/20:4) | 2.35±1.37 | 0.16±1.73 | 0.741 |
| PC(16:0e/20:4) | 0.20±0.89 | 0.15±1.10 | 0.758 |
| PC(36:4p) | 0.82±8.46 | 0.62±7.85 | 0.863 |
| PC(38:4p) | 0.43±10.2 | 0.68±10.8 | 0.868 |
| PC(40:7) | 0.78±3.87 | 0.84±5.02 | 0.936 |
| PC(16:0/22:4) | 0.75±3.46 | 0.77±4.47 | 0.970 |
| PC(33:1) | 0.38±2.46 | 0.37±2.06 | 0.978 |
| PC(37:4) | 0.42±3.21 | 0.42±5.87 | 0.996 |
| **PE** | | | |
| PE(16:0p/18:2) | 0.36±0.23 | -0.08±0.26 | 0.219 |
| PE(18:0/20:4) | 2.93±0.69 | 4.45±1.02 | 0.237 |
| PE(16:0/20:4) | 1.00±0.26 | 1.33±1.33 | 0.407 |
| PE(16:0p/20:4) | 1.36±0.72 | 0.07±0.74 | 0.534 |
| PE(18:0/22:6) | 0.53±0.18 | 0.95±0.29 | 0.235 |
| PE(16:0e/22:6) | 0.90±0.94 | 0.17±1.29 | 0.648 |
| PE(18:0p/22:6) | 0.67±0.38 | 0.68±0.50 | 0.987 |
| PE(18:0/18:2) | 2.85±0.53 | 3.80±0.69 | 0.292 |
| PE(16:0/18:2) | 0.95±0.27 | 1.33±0.24 | 0.296 |
| PE(16:0p/22:6) | 0.74±0.42 | 0.11±0.46 | 0.316 |
| PE(16:0/22:6) | 0.86±0.40 | 1.41±0.56 | 0.429 |
| PE(18:0/20:4) | 0.40±1.53 | 0.52±1.58 | 0.572 |
| PE(18:0p/20:4) | 0.18±1.42 | 0.11±1.27 | 0.699 |
| PE(18:1p/22:6) | 0.30±0.27 | 0.28±0.33 | 0.963 |
| **PS** | | | |
| PS(39:2) | 0.71±13.2 | 3.07±12.5 | 0.202 |
| PS(39:3) | -0.06±1.39 | -0.29±1.68 | 0.303 |
| PS(39:1) | 1.16±15.6 | 2.38±14.5 | 0.571 |
| PS(39:3) | 1.76±5.53 | 1.50±8.24 | 0.792 |
| PS(43:5) | 0.34±1.39 | 0.28±1.66 | 0.802 |
| PS(41:4) | 0.34±1.58 | 0.39±2.13 | 0.867 |
| PS(35:0) | 1.75±0.48 | 1.88±0.70 | 0.883 |
| PS(43:6) | 1.53±0.42 | 0.87±0.37 | 0.249 |
| PS(41:5) | 0.67±3.68 | 0.57±4.13 | 0.856 |
| **PI** | | | |
| PI(18:0/20:4) | 0.38±1.36 | 1.90±0.95 | 0.250 |
| **SphM** | | | |
| SphM(d18:0/16:0) | -0.49±0.17 | 0.14±0.28 | 0.065 |
| SphM(d16:0/18:1) | 0.87±5.44 | -0.99±8.88 | 0.078 |
| SphM(d42:1) | 0.56±4.56 | -0.21±4.06 | 0.214 |
| SphM(d18:1/18:3) | 0.14±1.58 | -0.13±2.18 | 0.331 |
| SphM(d34:2) | 0.52±4.59 | -0.17±5.88 | 0.356 |
| SphM(d40:1) | 0.30±7.33 | -0.27±8.52 | 0.608 |
| SphM(d32:1) | 0.10±2.97 | -0.10±3.56 | 0.667 |
| SphM(d18:0/18:2) | 0.44±0.58 | 0.35±0.67 | 0.919 |
| SphM(d18:1/18:0) | 0.09±1.05 | 0.09±1.54 | 0.992 |
| SphM(d34:0) | 0.44±3.05 | -0.28±3.07 | 0.101 |
| SphM(d34:1) | 7.23±65.9 | -9.08±85.4 | 0.136 |
| SphM(d22:0/20:3) | 0.39±1.30 | 0.11±2.08 | 0.271 |
| SphM(d41:1) | 0.47±3.99 | -0.16±4.22 | 0.282 |
| SphM(d38:2) | 0.29±2.71 | -0.13±3.63 | 0.355 |
| SphM(d33:1) | 0.19±2.28 | -0.04±1.87 | 0.436 |
| SphM(d36:1) | 1.15±10.8 | 0.06±10.0 | 0.464 |
| SphM(d38:1) | 0.18±5.03 | 0.61±5.46 | 0.563 |
| SphM(d20:0/18:2) | 0.03±0.25 | 0.18±0.33 | 0.715 |
| SphM(d42:2) | -0.12±11.8 | -0.58±14.1 | 0.802 |
| SphM(d42:3) | 0.52±9.10 | 0.25±9.71 | 0.841 |
| **ChE** | | | |
| ChE(18:1) | 0.32±6.57 | -0.33±7.87 | 0.531 |
| ChE(22:6) | -0.09±1.10 | 0.17±1.46 | 0.174 |
| ChE(18:2) | -76.3±57.1 | -0.92±46.1 | 0.366 |
| ChE(16:0) | 0.24±0.77 | -0.58±0.77 | 0.453 |
| ChE(20:3) | -0.14±1.16 | -0.14±1.55 | 0.525 |
| ChE(20:4) | 0.23±10.9 | -0.28±10.6 | 0.738 |
| CerG2(d18:1/16:0) | 0.47±0.47 | 0.11±0.56 | 0.625 |
| LPC(18:0) | -1.34±9.04 | -1.51±9.58 | 0.897 |

PC

PE

PS


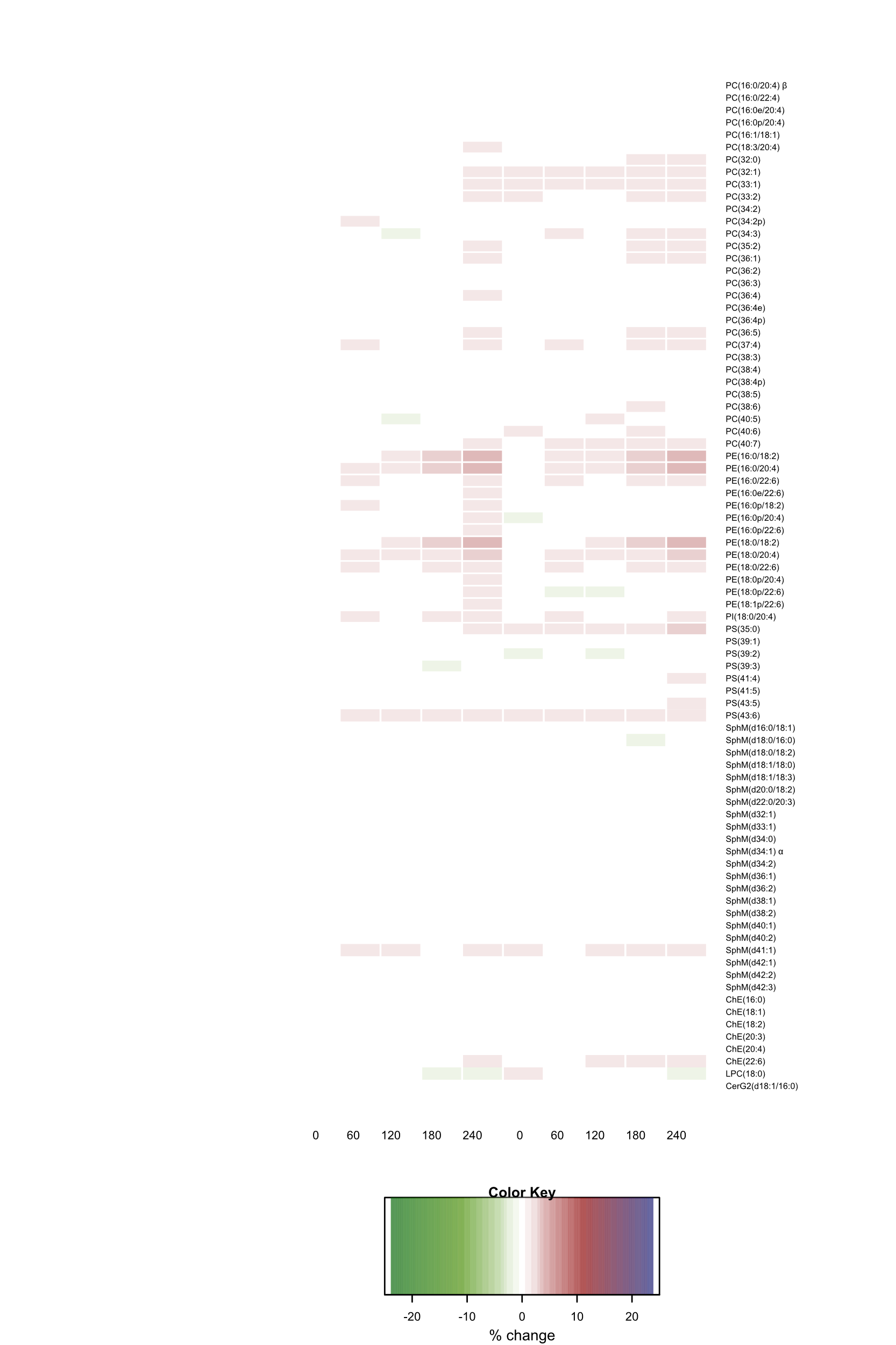


**Supplementary Figure 1: Heatmap of postprandial changes in individual phospholipids, sphingomyelins, and cholesterol ester species.** Values are presented as mean log fold % changes relative to concentrations at fasting SM (0 min); white represents a 0% change from SM baseline; red represents a 12.5% increase; blue represents and 25% increase; green represents a decrease; α denoted interaction time × milk with postprandial SM abundance greater than CM (*p*<0.05), while β denoted postprandial CM abundance is greater than SM.

Sheep

Cow

Milk

Time (min)

SphM


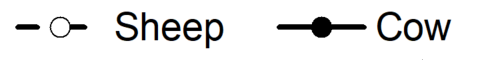


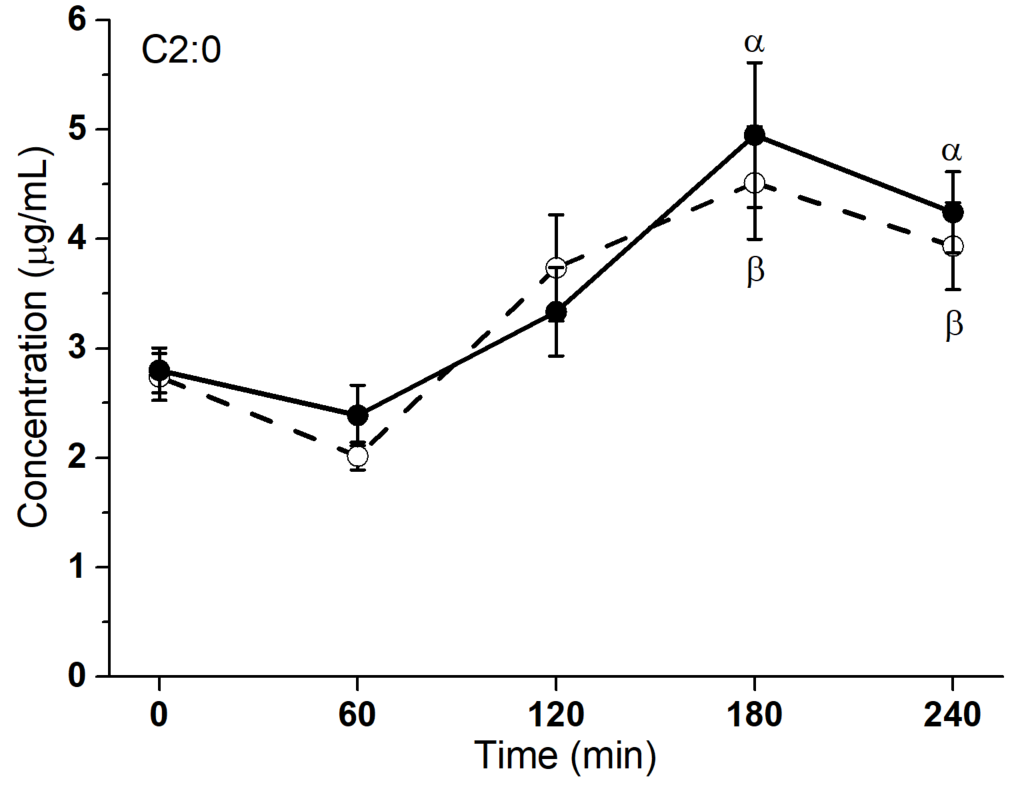

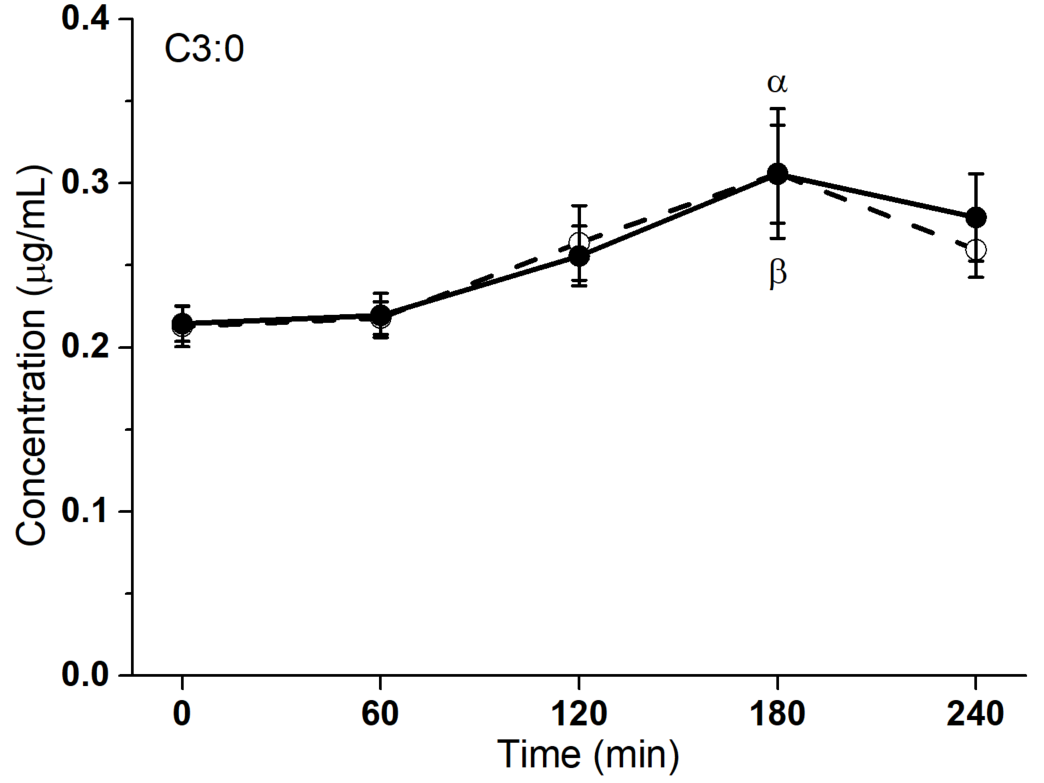

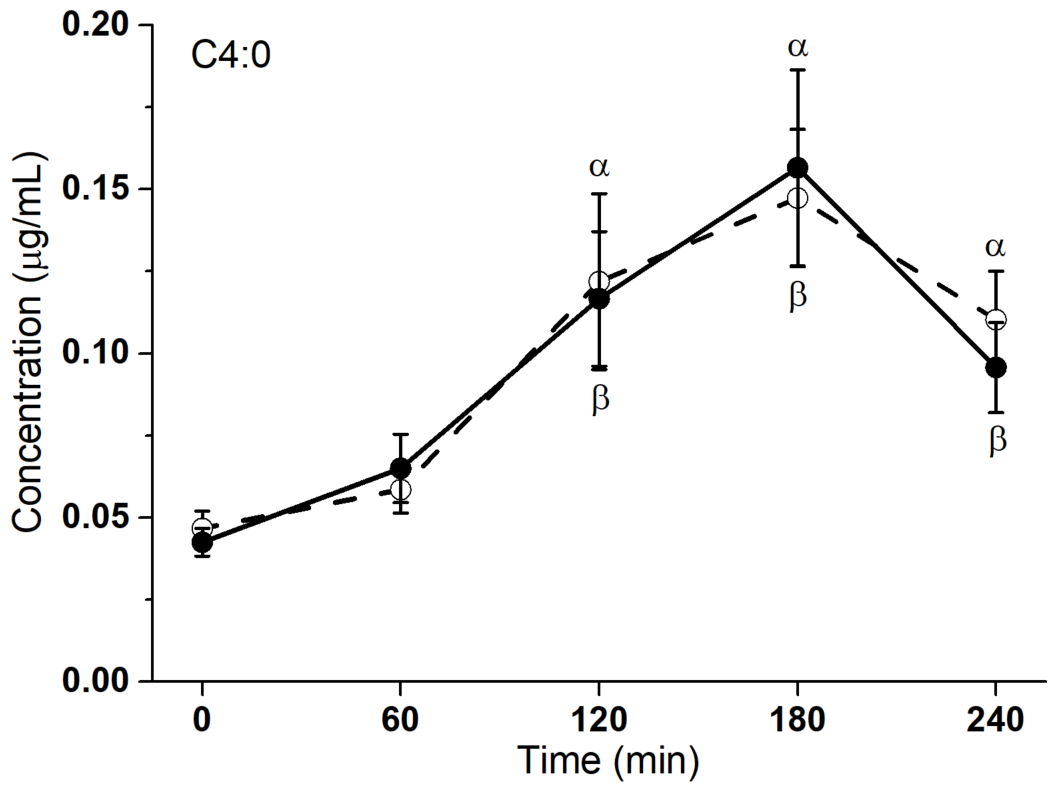


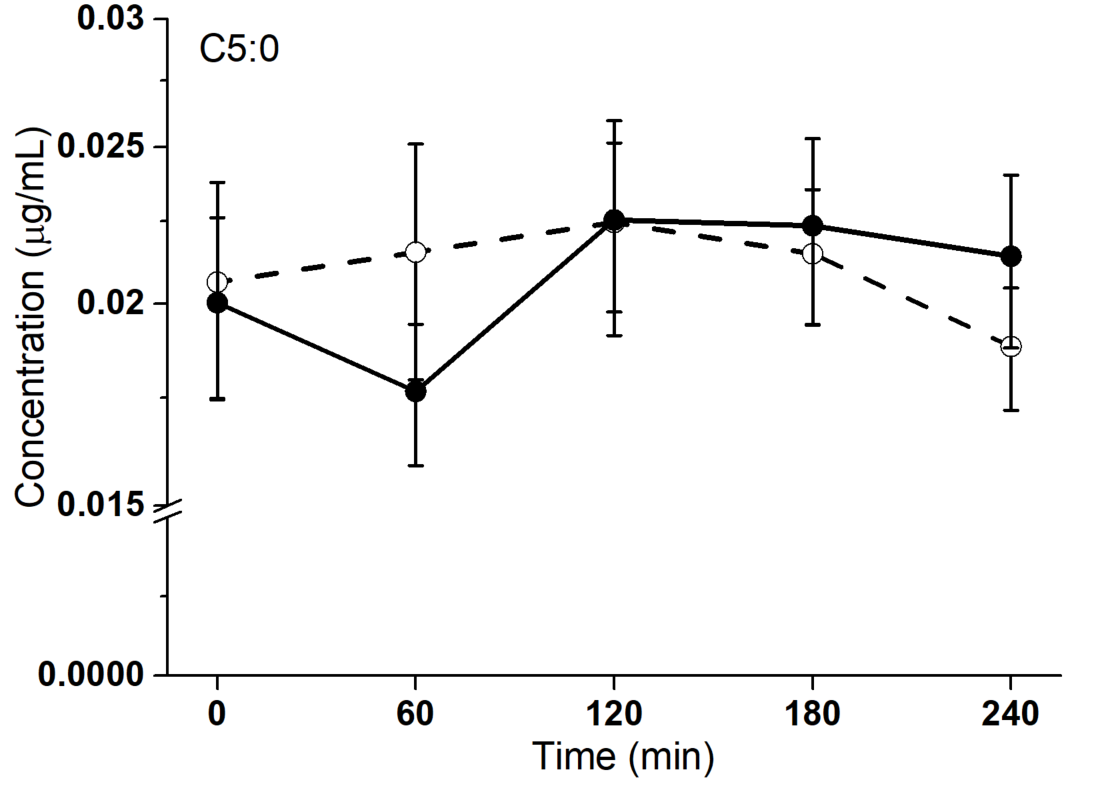

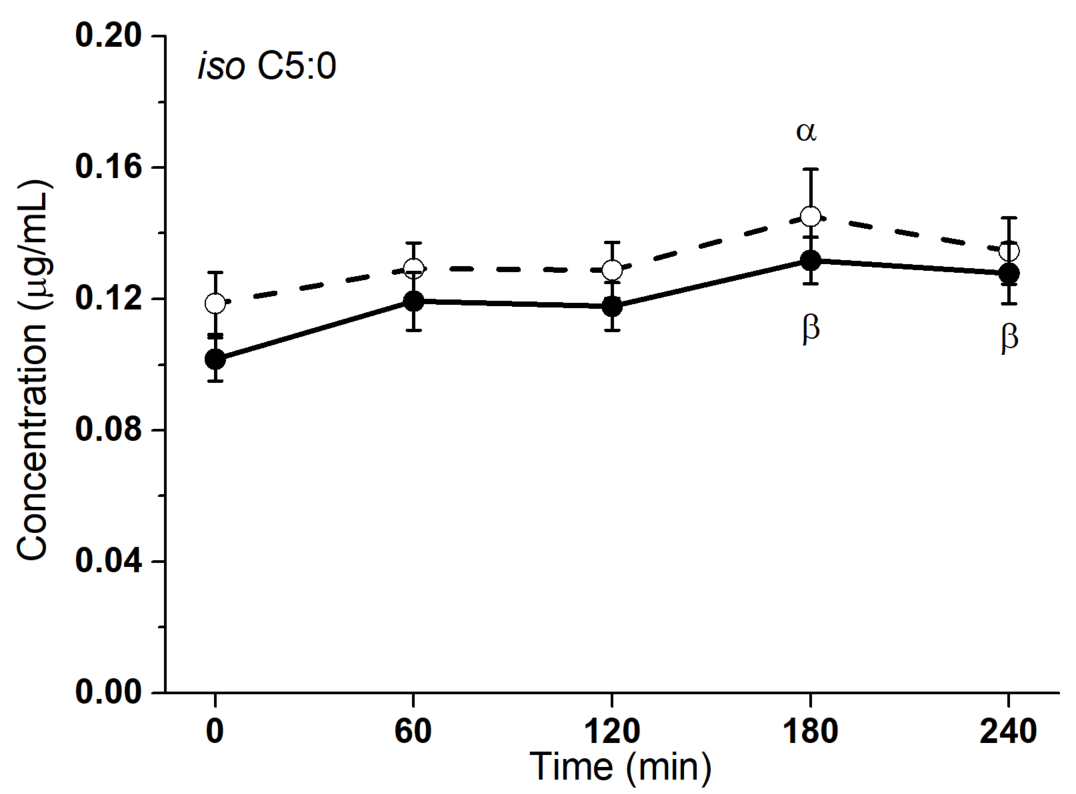

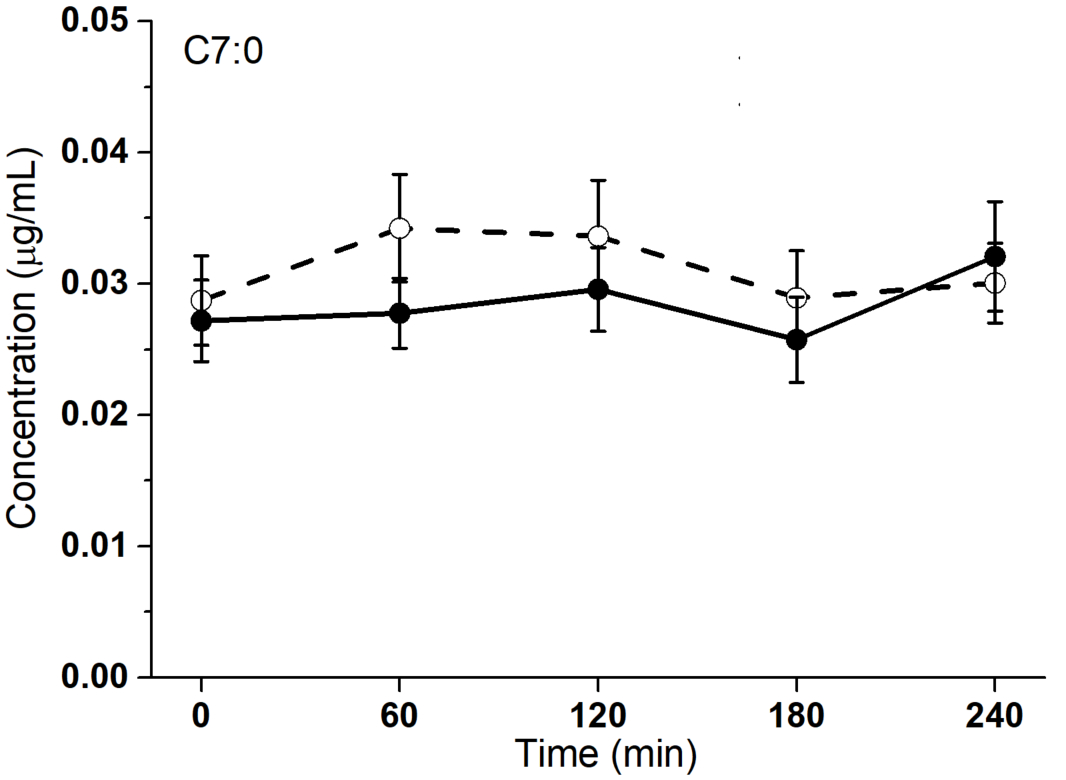


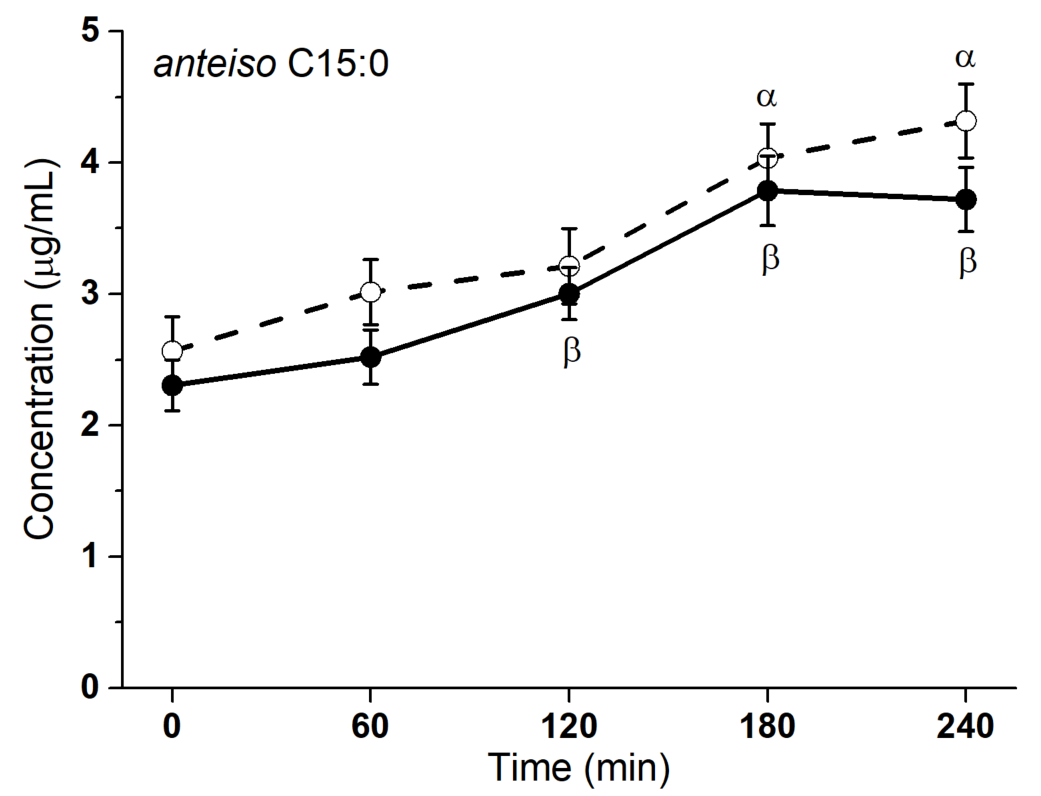

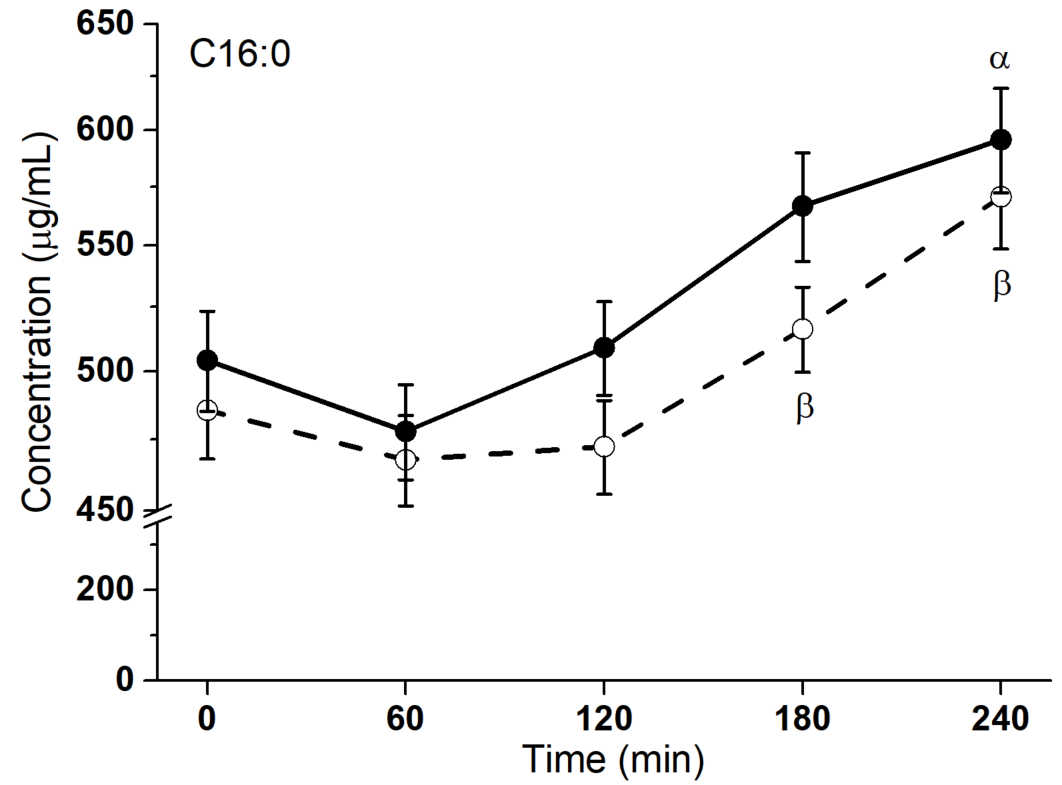

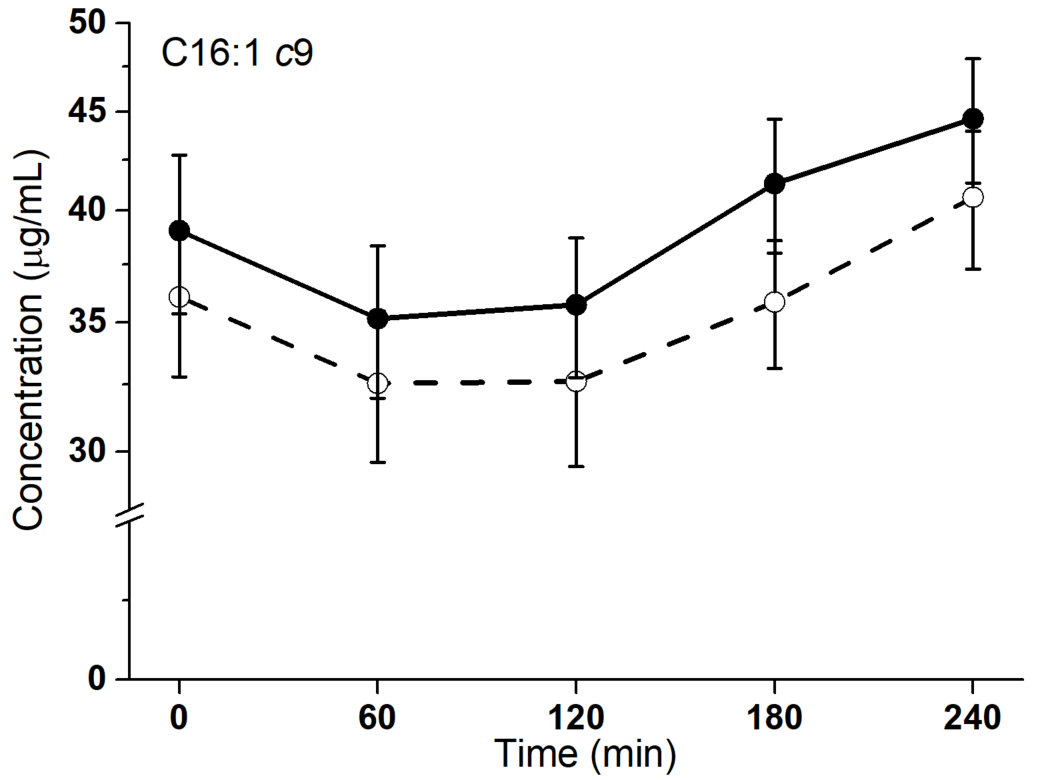


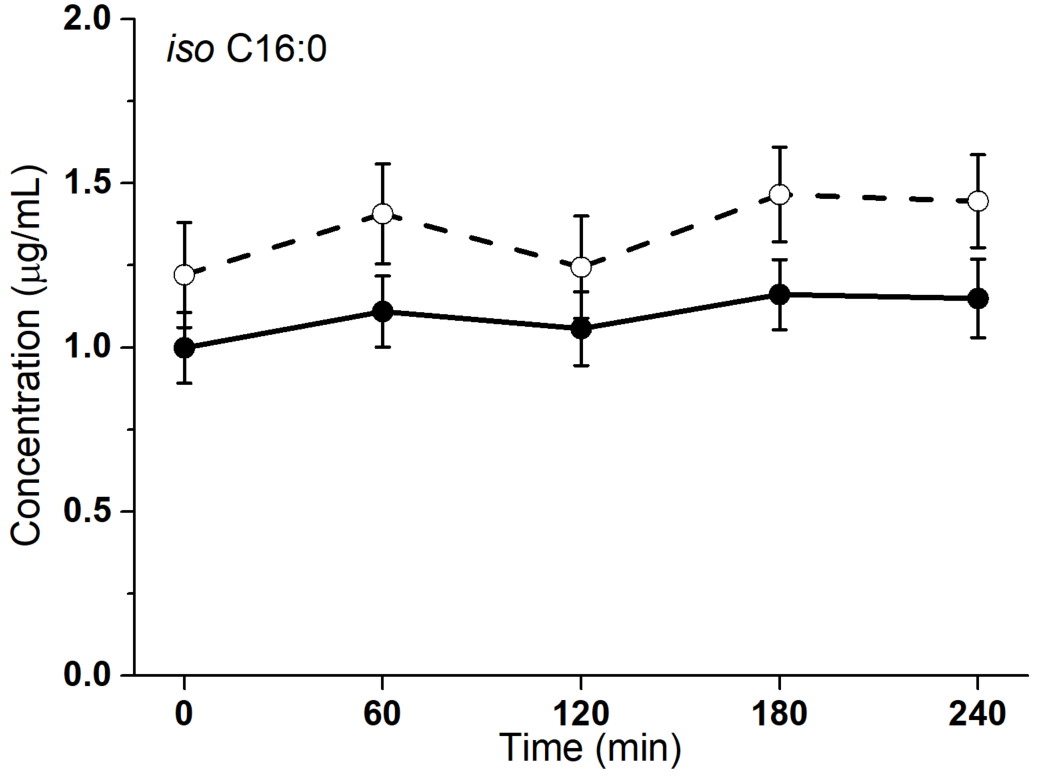

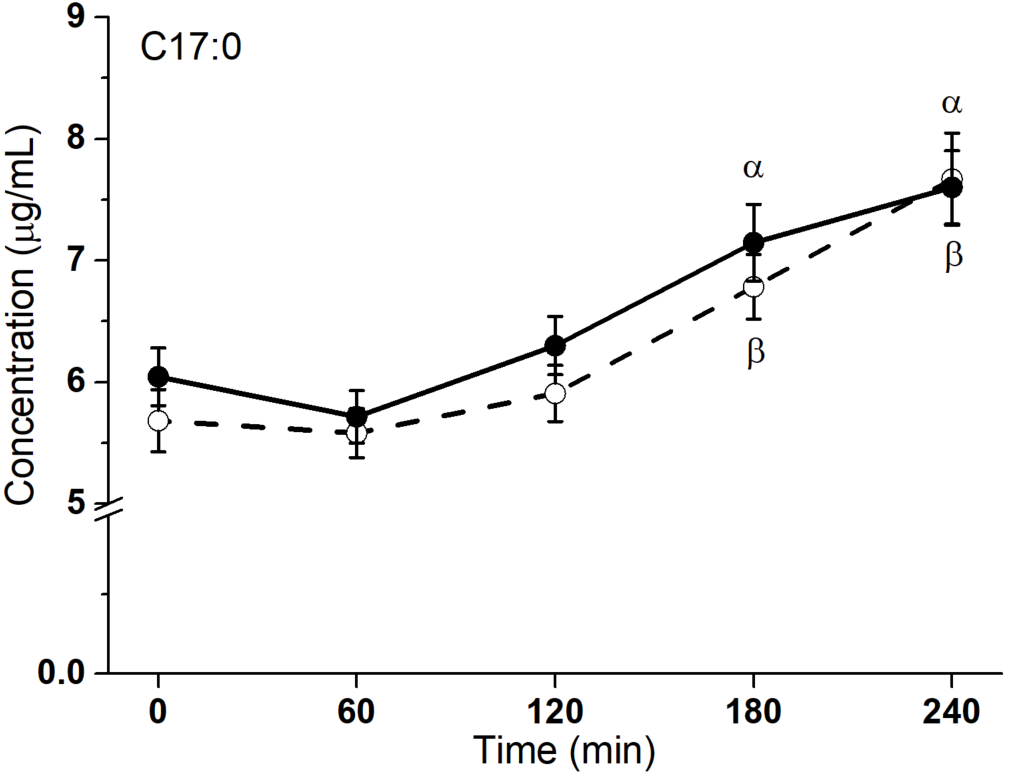

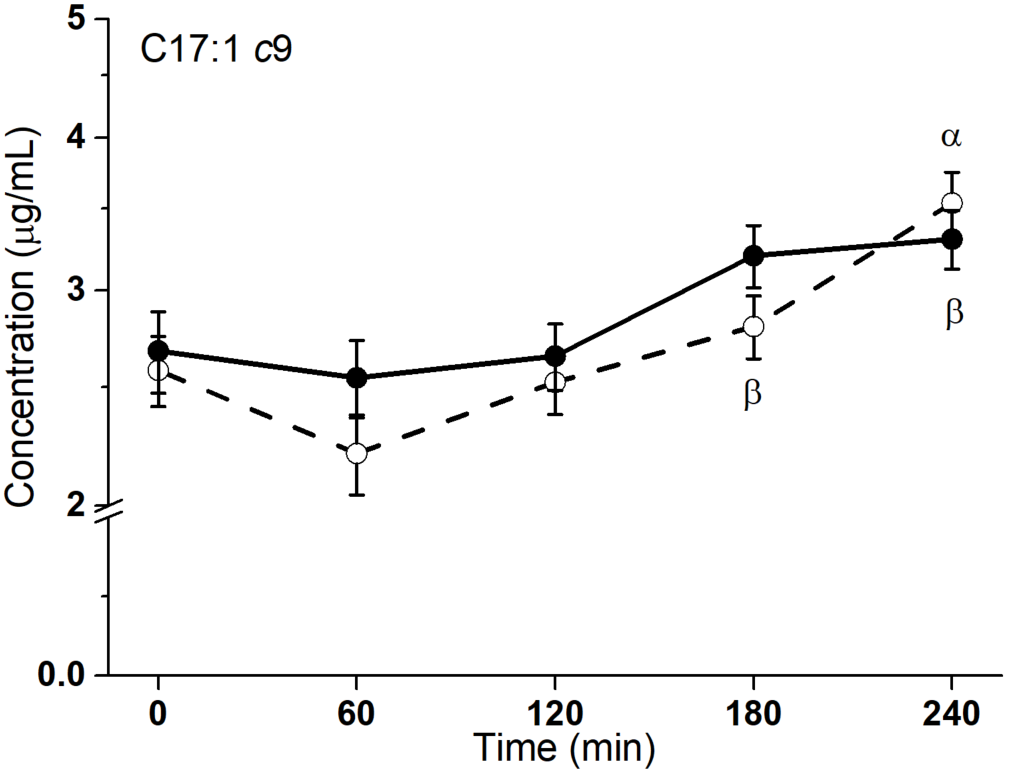


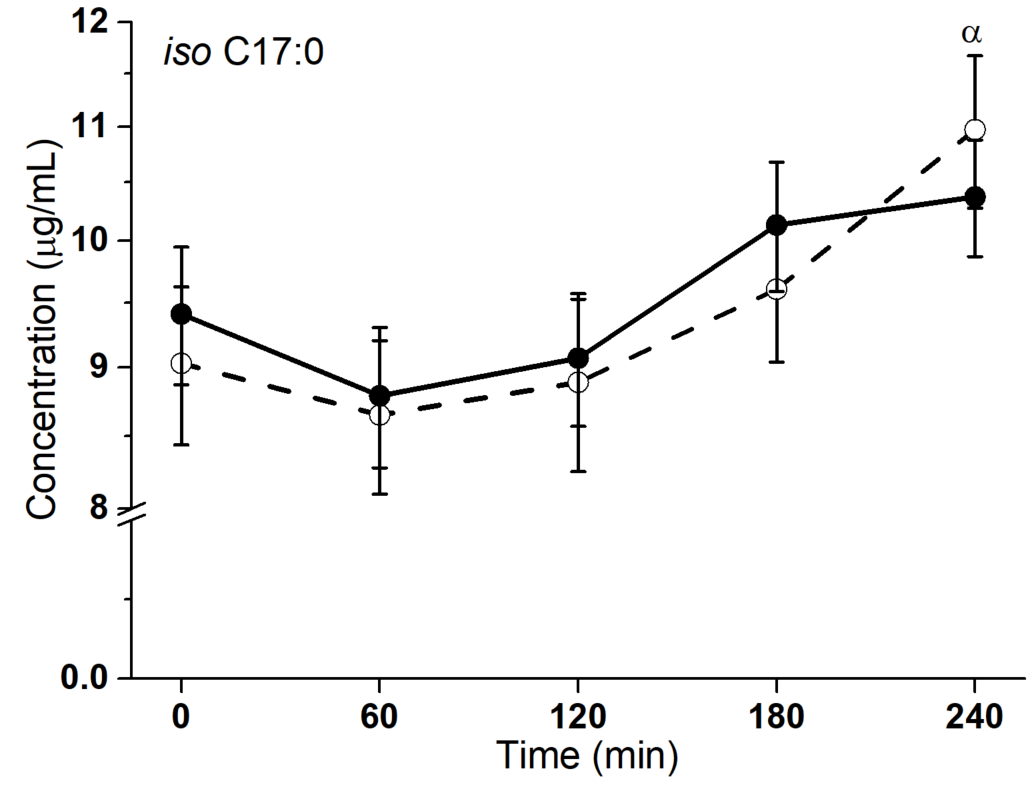

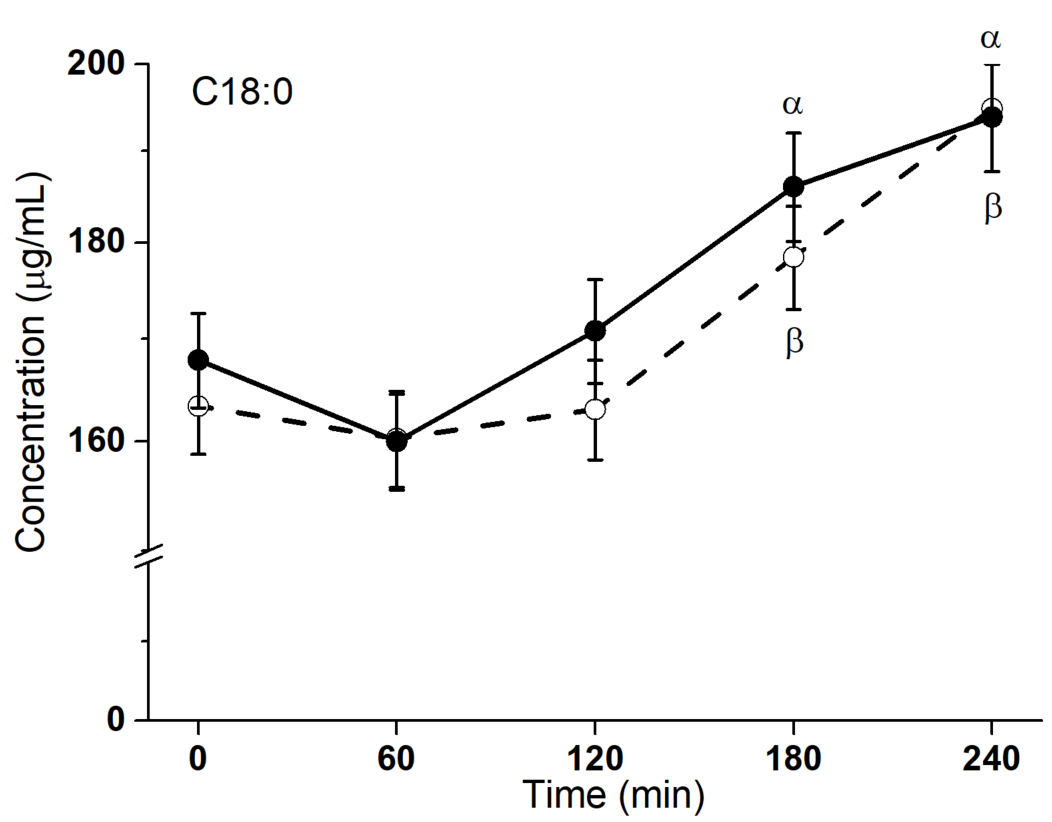

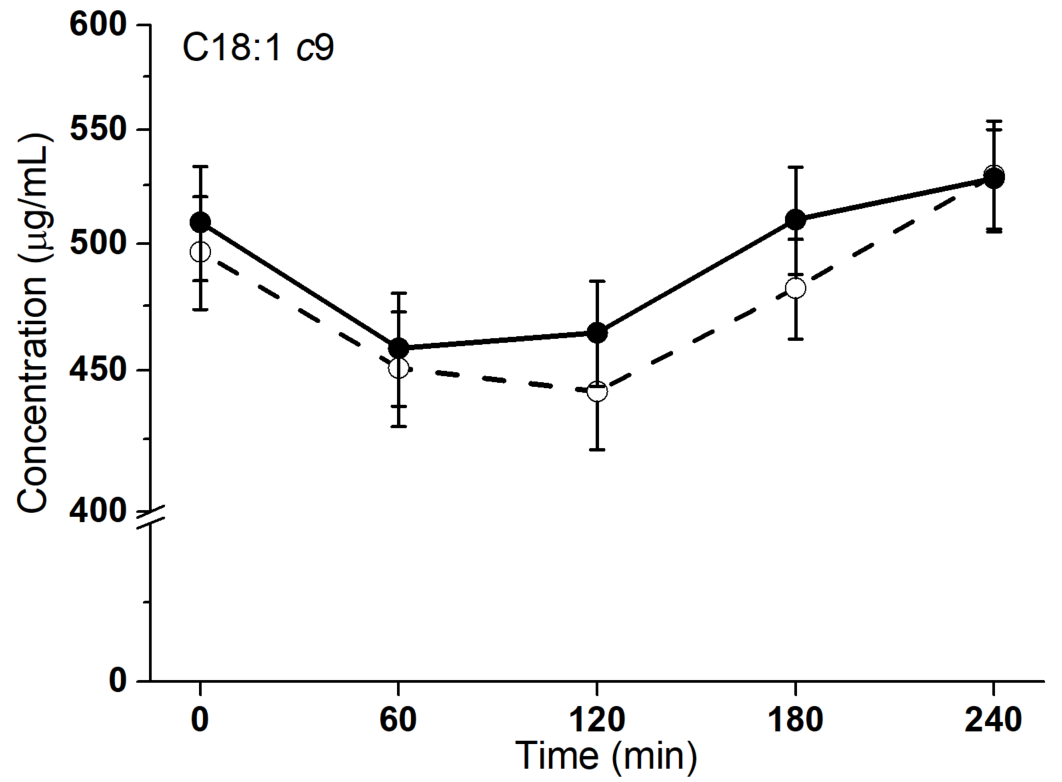


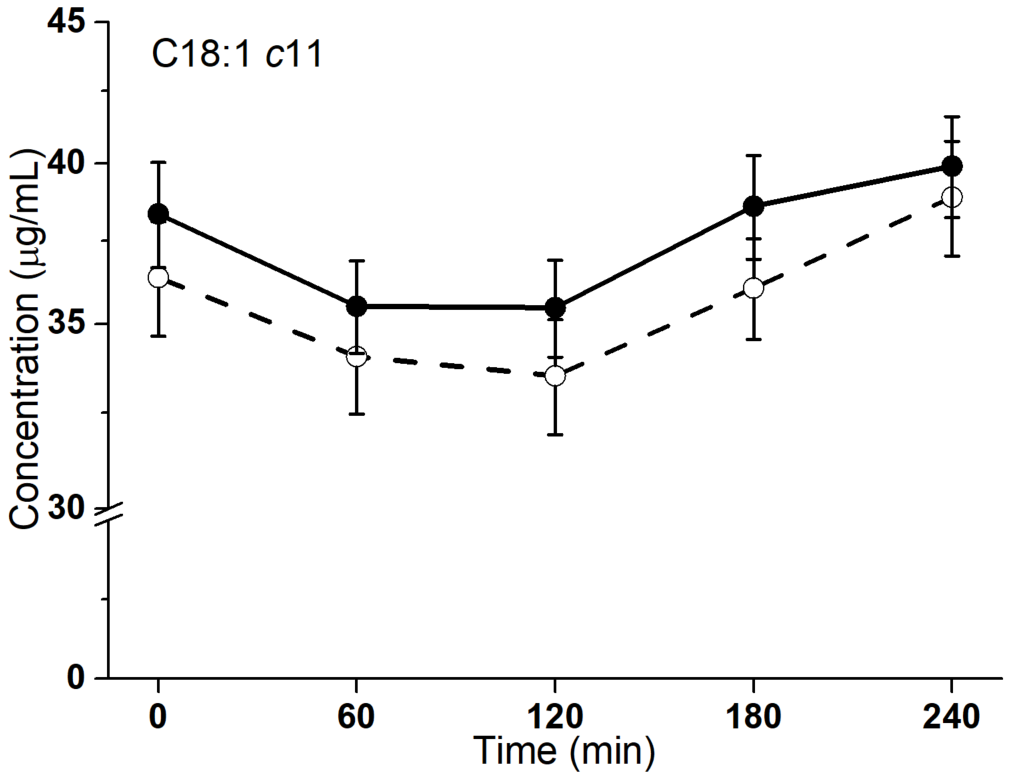

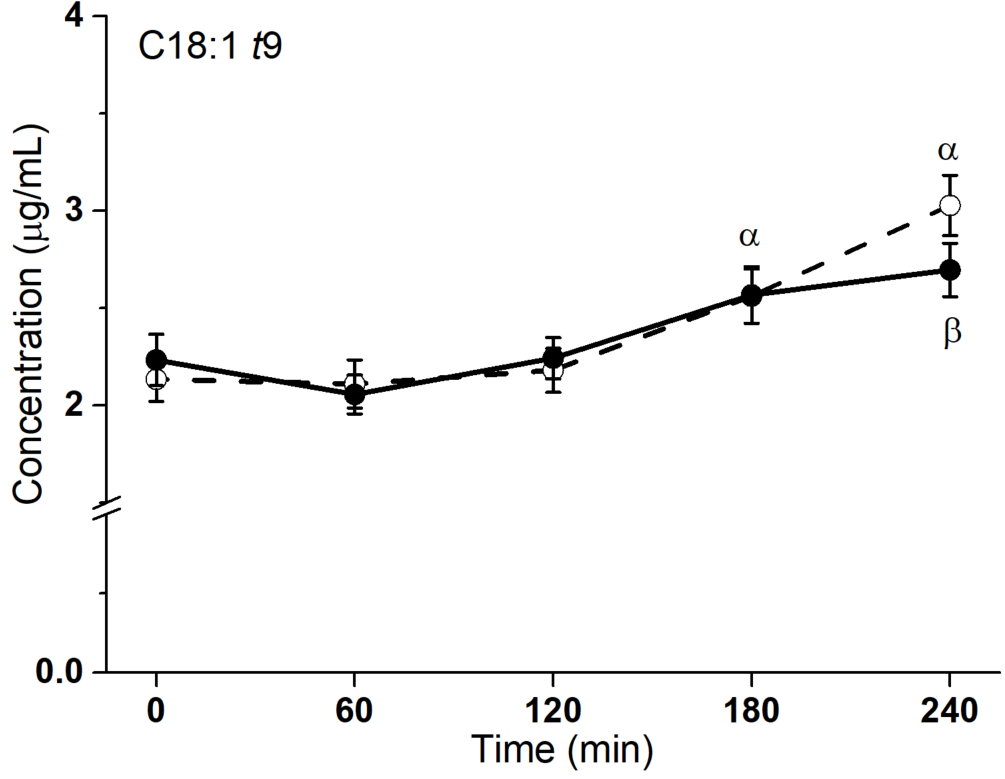

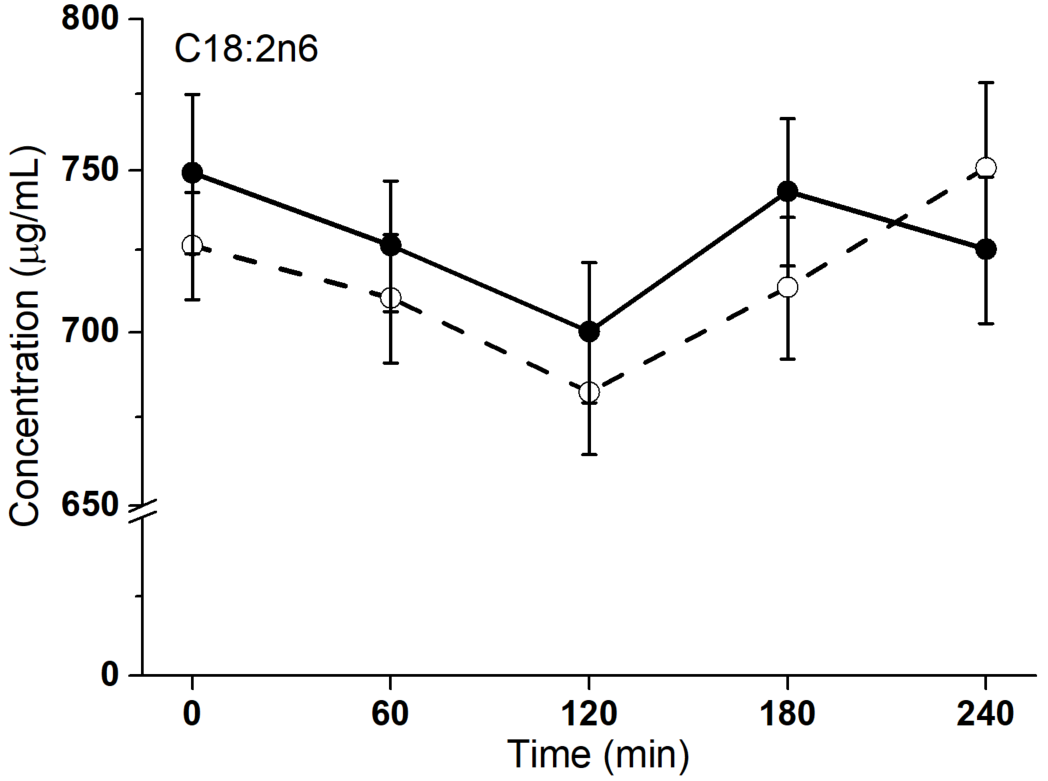


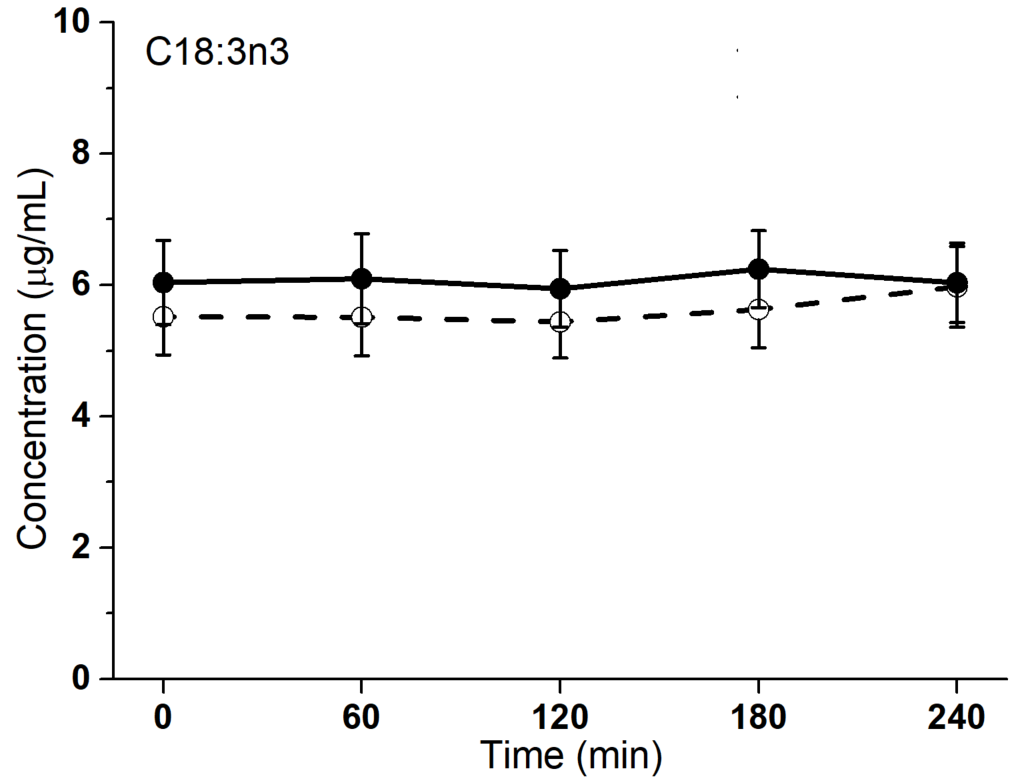

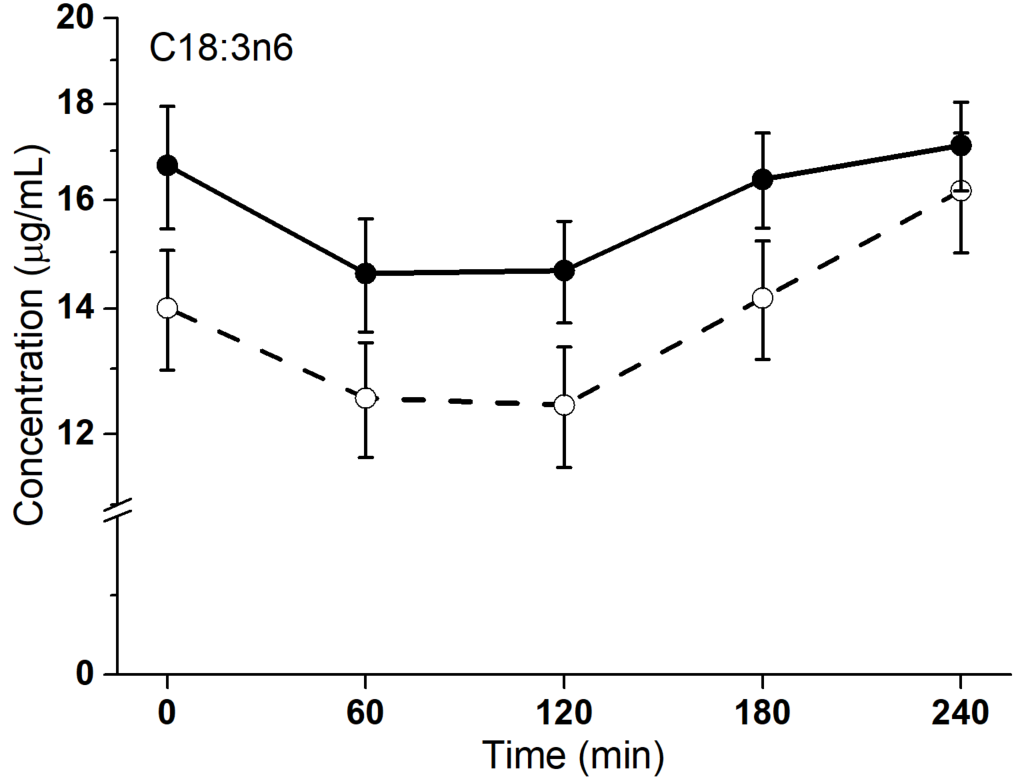

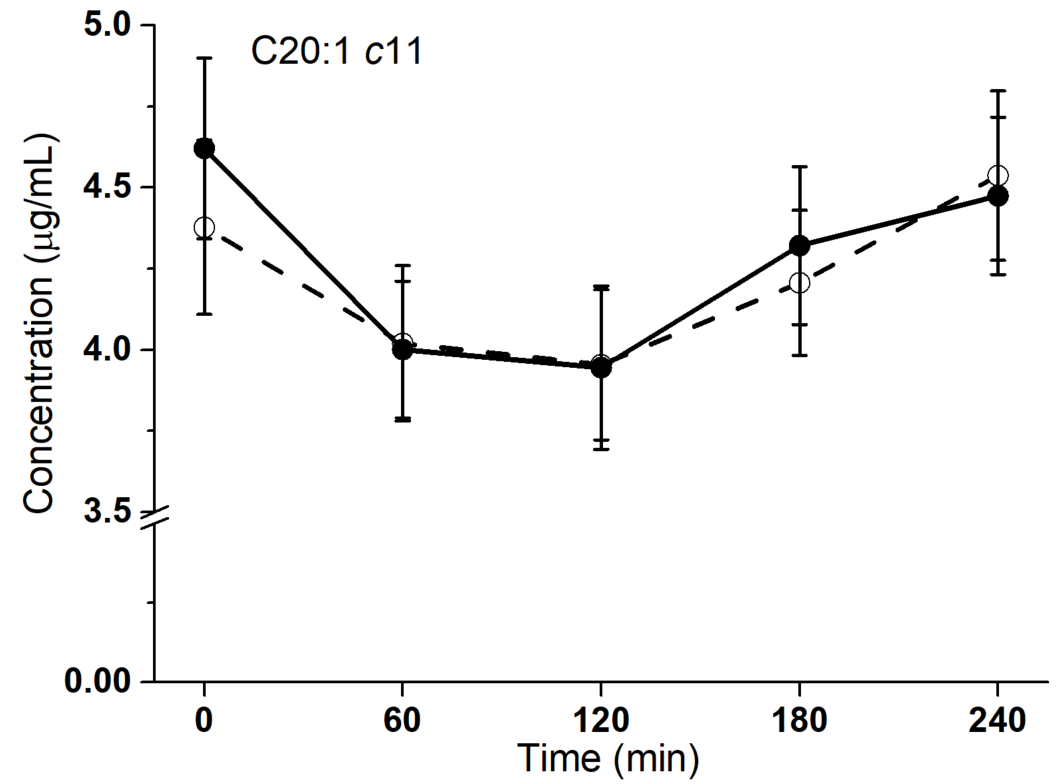

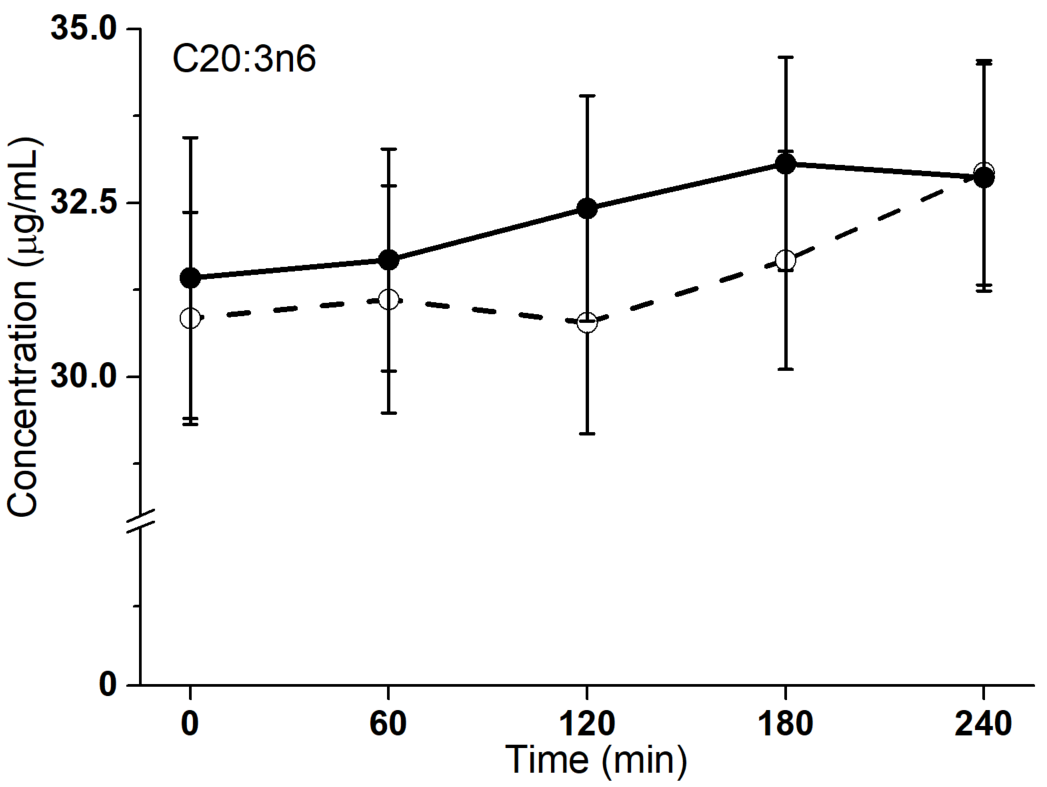

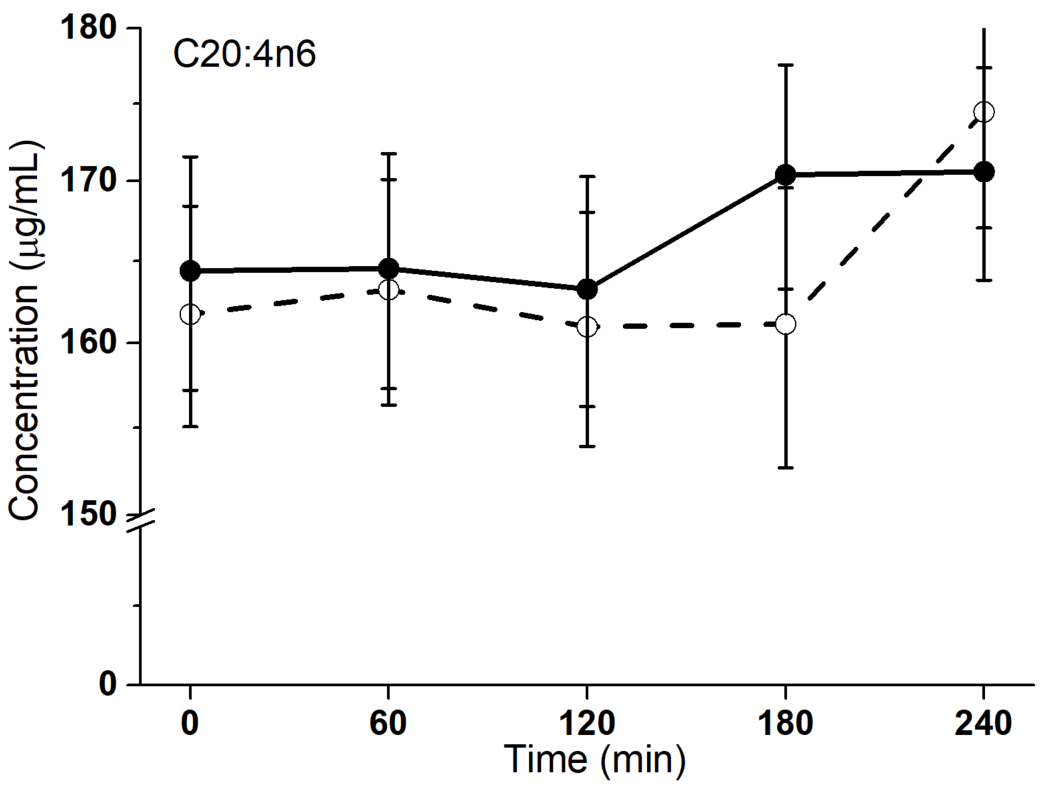

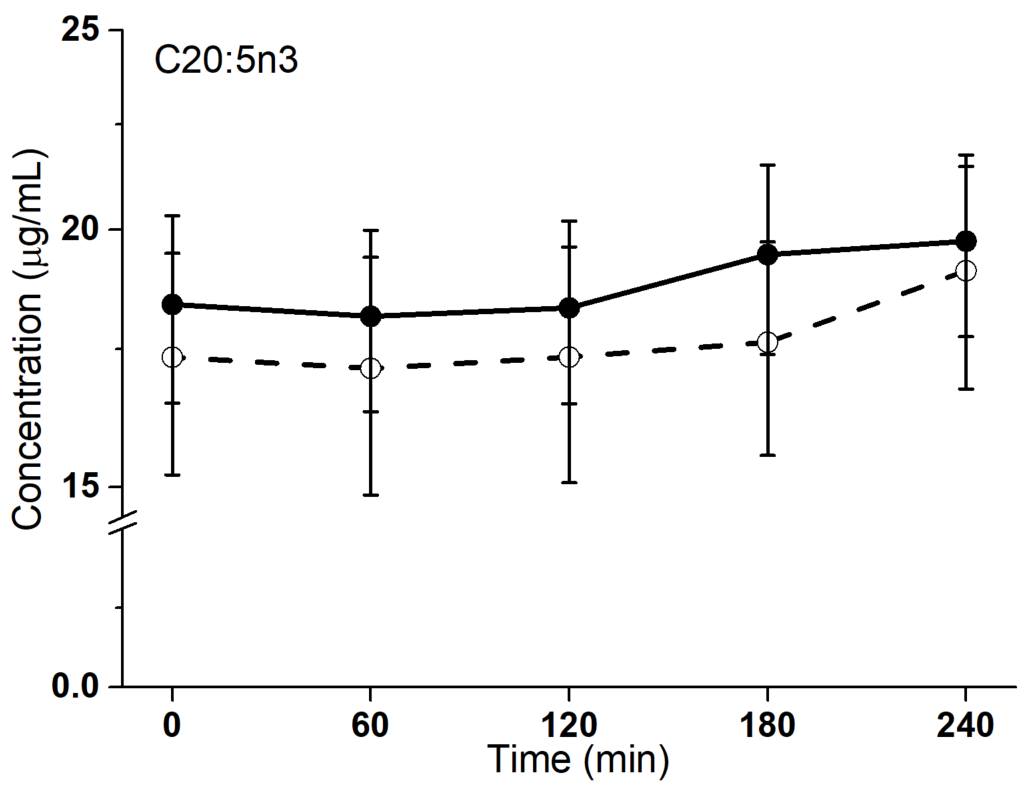


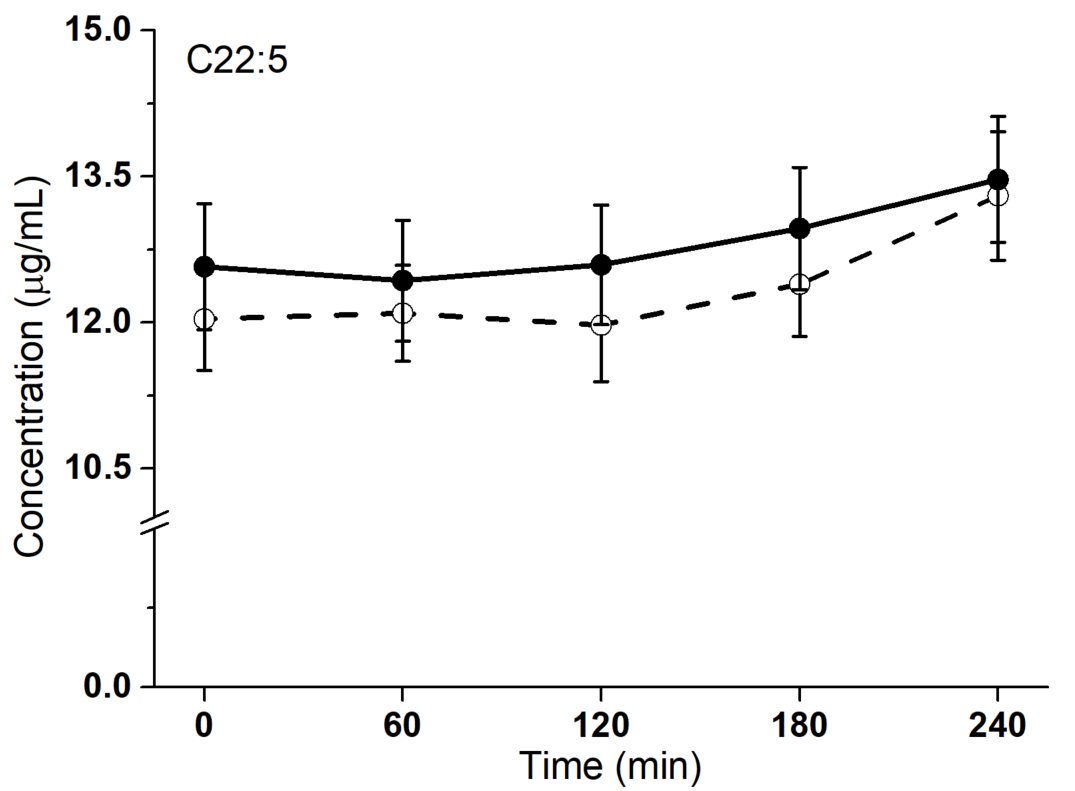

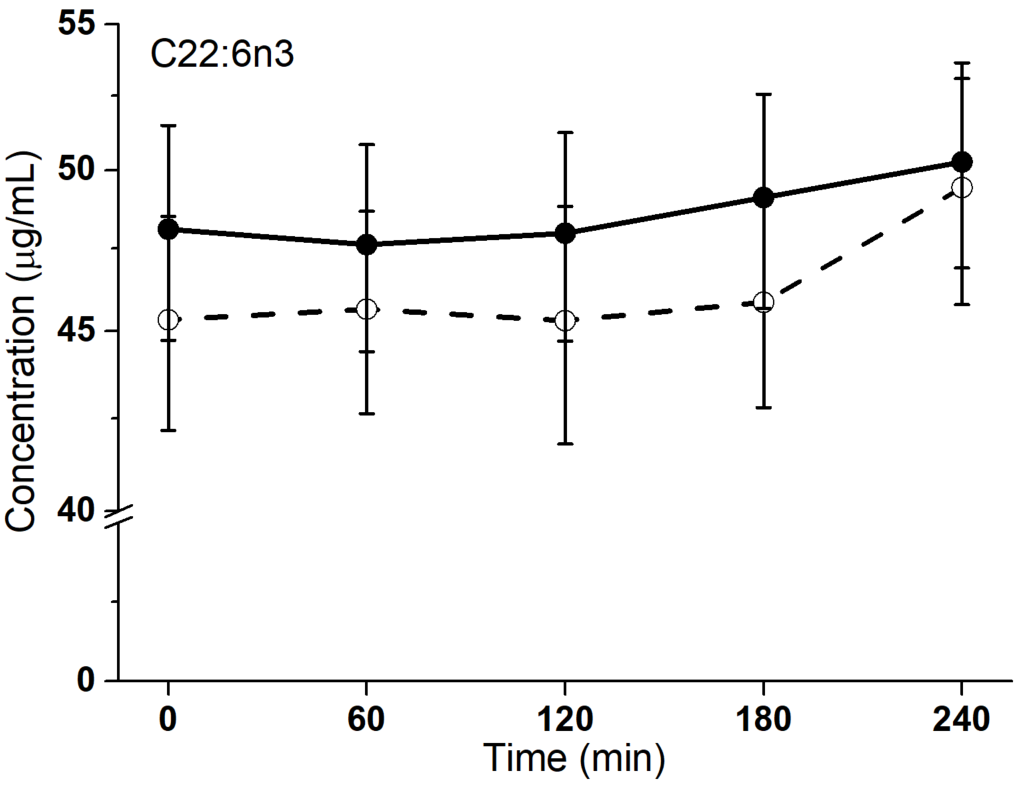


# Supplementary Figure 2: Postprandial changes in plasma fatty acids after sheep and cow milk ingestion. Values presented as means ± SEM; ^*^*p* < 0.05, ^**^*p* < 0.01, ^***^*p* < 0.001 denoted statistical significance (interaction time × milk) between sheep ( ) and cow ( ) milk; α and *β* denote significant changes (*p*<0.05) from baseline after sheep and cow milk ingestion, respectively (Sidak corrected post hocs).
